# Membrane Capacitive Memory Alters Spiking in Neurons Described by the Fractional-Order Hodgkin-Huxley Model

**DOI:** 10.1371/journal.pone.0126629

**Published:** 2015-05-13

**Authors:** Seth H. Weinberg

**Affiliations:** Virginia Modeling, Analysis and Simulation Center, Old Dominion University, 1030 University Boulevard, Suffolk, Virginia, USA; Plymouth University, UNITED KINGDOM

## Abstract

Excitable cells and cell membranes are often modeled by the simple yet elegant parallel resistor-capacitor circuit. However, studies have shown that the passive properties of membranes may be more appropriately modeled with a non-ideal capacitor, in which the current-voltage relationship is given by a fractional-order derivative. Fractional-order membrane potential dynamics introduce capacitive memory effects, i.e., dynamics are influenced by a weighted sum of the membrane potential prior history. However, it is not clear to what extent fractional-order dynamics may alter the properties of active excitable cells. In this study, we investigate the spiking properties of the neuronal membrane patch, nerve axon, and neural networks described by the fractional-order Hodgkin-Huxley neuron model. We find that in the membrane patch model, as fractional-order decreases, i.e., a greater influence of membrane potential memory, peak sodium and potassium currents are altered, and spike frequency and amplitude are generally reduced. In the nerve axon, the velocity of spike propagation increases as fractional-order decreases, while in a neural network, electrical activity is more likely to cease for smaller fractional-order. Importantly, we demonstrate that the modulation of the peak ionic currents that occurs for reduced fractional-order alone fails to reproduce many of the key alterations in spiking properties, suggesting that membrane capacitive memory and fractional-order membrane potential dynamics are important and necessary to reproduce neuronal electrical activity.

## Introduction

The properties of excitable cells and cell membranes have been studied for over a century, dating back to the studies of Weiss [1], Lapicque [2], and Nernst [3] in the early 1900s. These early studies established the now well-known inverse relationship between the strength of a stimulus necessary to elicit a spike (action potential) and the stimulus duration. Theoretical models were developed to explain the empirically-derived strength-duration curves: Representing the passive cell membrane as a parallel resistor-capacitor circuit, assuming *ideal* capacitive behavior, the membrane voltage *V*_*m*_ dynamics are given by

$$\begin{array}{c} C_{m}\frac{dV_{m}}{dt}+\frac{V_{m}}{R_{m}}=I_{app}\left(t\right), \end{array}$$ (1)

where *C*_*m*_ is membrane capacitance, *R*_*m*_ is membrane resistance, and *I*_*app*_ represents the applied stimulus current. This simple yet elegant membrane representation is the foundation for essentially all excitable cell models, originating with the Hodgkin-Huxley model [4], in which ionic currents are represented by time- and voltage-dependent conductances, in parallel with the membrane capacitance. Although minimal, the passive membrane model can predict a wide range of sub-threshold and threshold behavior. For example, if the applied current is a current step at time *t* = 0, i.e., *I*_*app*_(*t*) = *I*_*m*_ *u*(*t*) (where *u*(*t*) is the standard Heaviside function), then the solution of this ordinary differential equation,

$$\begin{array}{c} V_{m}\left(t\right)=I_{m}R_{m}(1-\exp(-t/\tau )), \end{array}$$ (2)

where membrane time constant *τ* = *R*_*m*_ *C*_*m*_ and the membrane is assumed to be initially uncharged (*V*_*m*_(0) = 0), can be rearranged to solve for the threshold current *I*_*t*_ necessary to elicit a spike for a stimulus of duration *d*, i.e., the strength-duration relationship,

$$\begin{array}{c} I_{t}=\frac{I_{rheo}}{1-\exp(-d/\tau )}, \end{array}$$ (3)

where rheobase current *I*_*rheo*_ = *V*_*t*_/*R*_*m*_ is the current threshold for an infinite duration stimulus and *V*_*t*_ is the membrane potential threshold. Eq 3 accurately reproduces the asymptotic behavior for long duration stimuli in the strength-duration curve observed in experiments. However, for short duration stimuli (*d* ≪ *τ*), Eq 3 is, to first-order, equivalent to an inverse scaling,

$$\begin{array}{c} I_{t}=I_{rheo}\tau d^{-1}, \end{array}$$ (4)

and early studies showed that experimental data did not well-fit this simple inverse scaling, *d* ^−1^. Rather, a general power law relation, *d* ^−*α*^, was more appropriate, with *α* ranging from 0.5 to 1 [5]. This was shown as early as 1933, when Cole notes Lapicque’s experimental data fits an average value of *α* = 0.656, while Ruston’s data fit values of *α* = 0.76 and 0.86 for warm and cold frog sciatic nerve, respectively [5].

Westerlund and Ekstram showed that Jacques Curie’s 1889 empirical law for current through capacitors and dielectrics [6] can be used to derive the capacitive current-voltage relationship for a *non-ideal* capacitor [7],

$$\begin{array}{c} I_{c}^{\alpha }=C_{m}^{\alpha }\frac{d^{\alpha }V_{c}}{dt^{\alpha }}, \end{array}$$ (5)

where 0 < *α* < 1, $C_{m}^{\alpha }$ is a fractional-order capacitance with units (amp/volt)⋅sec^*α*^, and, for now, the exact definition of the fractional-order derivative *d* ^*α*^ *V*_*c*_/*dt* ^*α*^ will be left ambiguous but will be clarified shortly.

Magin showed that incorporating this non-ideal capacitor into a fractional-order membrane model can reproduce the scaling of the strength-duration curve at both short and long durations [8]. Specifically, the fractional passive membrane dynamics are given by the fractional-order differential equation (cf. Eq 1)

$$\begin{array}{c} C_{m}^{\alpha }\frac{d^{\alpha }V_{m}}{dt^{\alpha }}+\frac{V_{m}}{R_{m}}=I_{m}u\left(t\right). \end{array}$$ (6)

**Fig 1:**
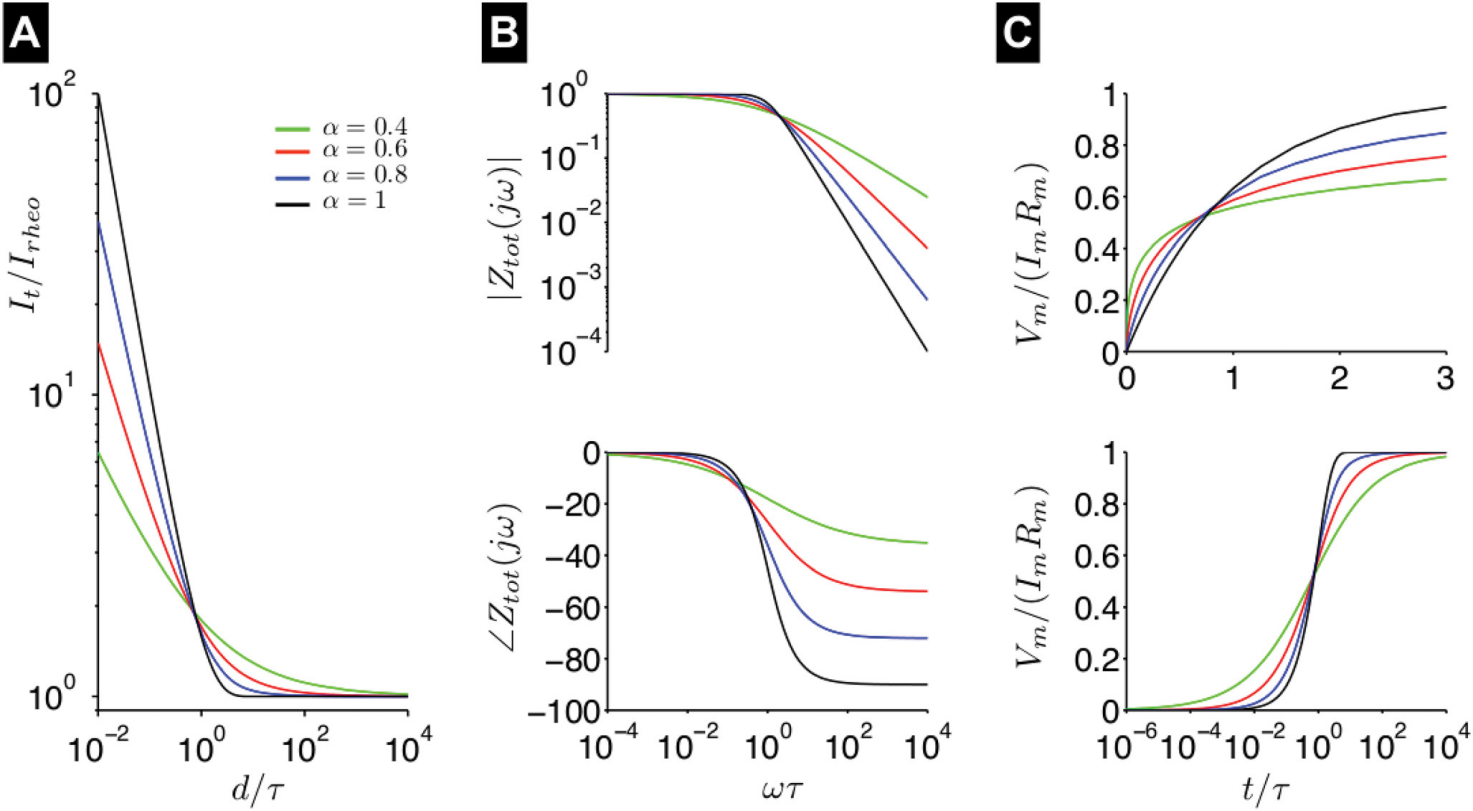
Properties of the fractional-order passive membrane. (A) Strength-duration curves, derived from the fractional passive membrane, are shown as a function of fractional-order *α* (Eq 9). (B) The magnitude 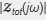 (top) and phase 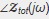 (bottom) of the complex impedance of the fractional-order passive membrane are shown as a function of the normalized frequency *ωτ*, for different values of *α*. (C) The normalized membrane potential *V*_*m*_/(*I*_*m*_ *R*_*m*_) response following a current step is shown as a function of normalized time *t*/*τ* on a linear (top) and logarithmic (bottom scale), for different values of *α*.

To our knowledge, the physiological source of such non-ideal capacitive behavior is not known, but we speculate may arise due to heterogeneities in the dielectric properties of the membrane, or more generally what is termed “capacitance dispersion” in electroanalytical chemistry [9]. As shown in S1 Text, Eq 6 can be solved via the Laplace transformation, with an analytical solution given by (cf. Eq 2)

$$\begin{array}{c} V_{m}\left(t\right)=R_{m}I_{m}[1-E_{\alpha ,1}(-{\left(t/\tau \right)}^{\alpha })], \end{array}$$ (7)

where $\tau^{\alpha }=R_{m}C_{m}^{\alpha }$, *E*_*α*,*β*_ is the two-parameter Mittag-Leffler function, which generalizes the exponential function, given by

$$\begin{array}{c} E_{\alpha ,\beta }\left(z\right)=\sum_{k=0}^{\infty }\frac{z^{k}}{\Gamma (\alpha k+\beta )}, \end{array}$$ (8)

and Γ(*x*) is the gamma function. Note that for *α* = 1, *E*_1,1_(*z*) = exp(*z*), and Eqs 2 and 7 are equivalent, as expected. Eq 7 can be rearranged, as before (cf. Eq 3),

$$\begin{array}{c} I_{t}=\frac{I_{rheo}}{1-E_{\alpha ,1}\left(-{\left(d/\tau \right)}^{\alpha }\right)}, \end{array}$$ (9)

and, importantly, yield a strength-duration relation that follows a general power law for short duration stimuli and asymptotic behavior for long duration stimuli (Fig 1A).

Despite these early studies which suggest that a non-ideal (or fractional-order) capacitive element may be more appropriate to represent passive membrane dynamics, essentially all excitable cell models assume an ideal capacitor, i.e., *α* = 1. In recent years, fractional-order dynamics have been shown to provide an improved description of many biological phenomena, including mechanical properties of viscoelastic tissue [10], the tissue-electrode interface [11], pharmacokinetics of drug delivery and absorption [12–14], and anomalous calcium subdiffusion in microdomains [15]. While it is clear that fractional-order membrane potential *V*_*m*_ dynamics will alter the *passive* response to sub-threshold stimuli, it is not obvious if fractional-order dynamics will alter the properties of the *active* neuron and response to super-threshold stimuli, given the bi-directional coupling between *V*_*m*_ and ionic currents. In this study, we will investigate fractional-order *V*_*m*_ dynamics in the classical Hodgkin-Huxley model [4] and test the hypothesis that fractional-order *V*_*m*_ dynamics influence the properties of the *active* neuron across multiple spatial scales. In this study, we provide a brief background on the mathematics of fractional calculus and elaborate on and illustrate some properties of the fractional-order passive membrane, introduce the fractional-order Hodgkin-Huxley model, and characterize properties of neuronal spikes. We study the properties of sub-threshold and spike propagation in a nerve axon, characterizing the passive and active cable, respectively. Finally, we study the dynamical properties of a network of fractional-order neurons. We conclude with a discussion of our results.

## Background on fractional calculus

### Definitions of the fractional-order derivative

Our brief background on fractional calculus presented here is by no means complete. For further details, methods, and applications of fractional derivatives and differential equations, we suggest the monographs by Podlubny [16] and Magin [17]. The definition of a fractional-order derivative is not immediately obvious, and indeed, multiple definitions exist. The classical definition of the fractional derivative of order *α*, known as the Riemann-Liouville fractional derivative, denoted ${}_{0}D_{t}^{\alpha }$ and given by

$$\begin{array}{c} {}_{0}D_{t}^{\alpha }y\left(t\right)=\frac{1}{\Gamma (m-\alpha )}\frac{d^{m}}{dt^{m}}[\int_{0}^{t}\frac{y(\tau )}{{\left(t-\tau \right)}^{\alpha +1-m}}d\tau ], \end{array}$$ (10)

where *m*−1 < *α* < *m* (i.e., *m* is the first positive integer larger than *α*), follows from the definition of the fractional integral.

Note that the subscripts 0 and *t* on ${}_{0}D_{t}^{\alpha }$ indicate limits for the fractional integral and highlight the fact that, in contrast with integer-order derivatives, the fractional-order derivative depends on the *previous history* of the function and is not strictly a *local* property. An alternative definition was given by Caputo as

$$\begin{array}{c} {}_{0}^{C}D_{t}^{\alpha }y\left(t\right)=\frac{1}{\Gamma (m-\alpha )}\int_{0}^{t}\frac{y^{\left(m\right)}\left(t\right)}{{\left(t-\tau \right)}^{\alpha +1-m}}d\tau , \end{array}$$ (11)

where *y* ^(*m*)^(*t*) is the *m* ^*th*^-order derivative with respect to time. Note that both definitions involve fractional-order integration and integer-order differentiation, with the order of the two operations different. However, these definitions are not equivalent, except in the case that all initial conditions are equal to 0. This is analogous to the integer-order operators, integration and differentiation, not being commutative and differing by a constant. In this case, the difference is a function of time. The appropriate choice of representation will depend on the situation being modeled. However, the Caputo definition is often preferable for modeling physical processes, since initial conditions of fractional differential equations using the Caputo definition are specified in terms of integer-order derivatives. In contrast, fractional differential equations using the Riemann-Liouville definition requires initial conditions specified in terms of fractional-order derivatives, which typically have vague physical interpretation.

### Numerical methods

We consider one more definition for the fractional-order derivative, the Grunwald-Letnikov fractional derivative, which will lead to a simple numerical method for integrating a fractional differential equation [18]. Consider the well-known definition of the first-order derivative:

$$\begin{array}{c} y^{\prime }\left(t\right)=\lim_{\Delta t\to 0}\frac{y(t)-y(t-\Delta t)}{\Delta t}. \end{array}$$ (12a)

Applying this definition again gives us the second-order derivative,

$$\begin{array}{c} y^{\prime \prime }\left(t\right)=\lim_{\Delta t\to 0}\frac{y(t)-2y(t-\Delta t)+y(t-2\Delta t)}{{\left(\Delta t\right)}^{2}} \end{array}$$ (12b)

and, again, the third derivative,

$$\begin{array}{c} y^{\prime \prime \prime }\left(t\right)=\lim_{\Delta t\to 0}\frac{y(t)-3y(t-\Delta t)+3y(t-2\Delta t)-y(t-3\Delta t)}{{\left(\Delta t\right)}^{3}}. \end{array}$$ (12c)

By induction, the general *n* ^*th*^-derivative is given by

$$\begin{array}{ccc} y^{\left(n\right)}\left(t\right) & = & \lim_{\Delta t\to 0}\frac{1}{{\left(\Delta t\right)}^{n}}\sum_{k=0}^{n}{\left(-1\right)}^{k}(\begin{array}{c} n\\ k \end{array})y\left(t-k\Delta t\right)\\ & = & \lim_{\Delta t\to 0}\frac{1}{{\left(\Delta t\right)}^{n}}\sum_{k=0}^{n}{\left(-1\right)}^{k}\frac{\Gamma (n+1)}{\Gamma (k+1)\Gamma (n-k+1)}y\left(t-k\Delta t\right), \end{array}$$ (12d)

where $\left(\begin{array}{c} n\\ k \end{array}\right)=n!/(k!(n-k)!)$ is the usual notation for the binomial coefficients, and the second equality follows from the gamma function relation Γ(*n*) = (*n*−1)!. The use of the gamma function allows one to extend the derivative definition from integer-order values of *n* to arbitrary fractional-order.

It can be shown that the Grunwald-Letnikov definition is equivalent to the Riemann-Liouville definition [16]. In this study, we will consider the fractional-order Hodgkin-Huxley model initially at rest (*V*_*m*_(0) = 0), and thus all three definitions are equivalent. To illustrate the numerical integration scheme, consider a generic fractional differential equation with zero-initial conditions

$$\begin{array}{c} {}_{0}D_{t}^{\alpha }y\left(t\right)=f\left(t,y\right). \end{array}$$ (13)

Applying a finite-difference scheme to Eq 13 using Eq 12d, we have

$$\begin{array}{c} \frac{1}{{\left(\Delta t\right)}^{\alpha }}(y_{n+1}-\sum_{k=1}^{n+1}c_{k}^{\alpha }y_{n+1-k})=f\left(t_{n},y_{n}\right), \end{array}$$ (14a)

where

$$\begin{array}{c} c_{k}^{\alpha }={\left(-1\right)}^{k-1}\frac{\Gamma (\alpha +1)}{\Gamma (k+1)\Gamma (\alpha -k+1)} \end{array}$$ (14b)

Rearranging Eq 14a, an explicit numerical scheme is given by

$$\begin{array}{c} y_{n+1}=\sum_{k=1}^{n+1}c_{k}^{\alpha }y_{n+1-k}+{\left(\Delta t\right)}^{\alpha }f\left(t_{n},y_{n}\right), \end{array}$$ (15a)

where the $c_{k}^{\alpha }$ terms can be computed recursively using

$$\begin{array}{c} c_{k}^{\alpha }=(1-\frac{1+\alpha }{k})c_{k-1}^{\alpha } \end{array}$$ (15b)

and $c_{1}^{\alpha }=\alpha$. Thus, the $c_{k}^{\alpha }$ terms represent a “weighting” of previous history or memory on the current state of the system, a consequence of the non-locality of the fractional derivative. Values of $c_{k}^{\alpha }$ are shown in S1 Text for different values of *α* (Fig. A in S1 Text). For *α* = 1, all $c_{k}^{\alpha }$ terms beyond *k* = 1 are equal to 0, and Eq 15 is equivalent to the forward Euler method. Importantly, $c_{k}^{\alpha }$ decreases as *k* decreases, as the current value in the numerical integration depends increasingly less on earlier system states. However, as *α* decreases, $c_{k}^{\alpha }$ decreases more slowly, i.e., there is a larger dependence on the system history. Note that discretization of the Grunwald-Letnikov definition can be used to solve systems with non-zero initial conditions by including an additional term in the numerical scheme above [18].

## Fractional-order neuron model

### Fractional-order passive membrane properties

Before investigating the influence of fractional-order *α* on the properties of the active neuron, it is instructive to consider the response of the fractional-order passive membrane. We first consider the passive response to a sinusoidal current input of varying frequencies, which allows for standard circuit analysis using complex impedances. For the standard passive membrane patch, the resistor complex impedance is strictly real, 𝓩_*r*_ = *R*_*m*_. An ideal capacitor complex impedance is strictly imaginary, ${𝓩}_{c}=1/(j\omega C_{m})=1/(\omega C_{m})\exp(-\frac{\pi }{2}j)$, while a non-ideal capacitor complex impedance is given by ${𝓩}_{c}^{\alpha }=1/{\left(j\omega C_{m}\right)}^{\alpha }=1/{\left(\omega C_{m}\right)}^{\alpha }\exp(-\frac{\pi }{2}\alpha j)$, where *α* = 1 refers to an ideal capacitor and *α* = 0 refers to a pure resistor. Such a non-ideal capacitor is often called a *constant phase element*. The magnitude and phase of the total impedance for the passive membrane, i.e. the parallel resistor-capacitor circuit,

$$\begin{array}{c} {𝓩}_{tot}=\frac{1}{{𝓩}_{r}^{-1}+{\left({𝓩}_{c}^{\alpha }\right)}^{-1}}=\frac{R_{m}}{1+R_{m}{\left(j\omega C_{m}\right)}^{\alpha }}, \end{array}$$ (16)

are shown as a function of frequency *ω* for different values of *α* in Fig 1B. For all values of *α*, the total impedance magnitude approaches 1 at low frequencies and decreases at high frequencies, i.e., the membrane acts as a low-pass filter. However, for small *α*, the drop-off at high frequencies is reduced, i.e., the slope (on a log-log scale) is smaller, reflecting the power law relation present at short time scales. Further, the phase angle observed for high frequencies varies with *α*, as the phase approaches −90° for *α* = 1 and −45° for *α* = 0.5. From Eq 16, it can be shown that the phase at high frequencies is given by $-\frac{\pi }{2}\alpha$. Early studies by Cole and colleagues measured phases corresponding to *α* values between 0.61 and 0.86, consistent with measurements based on strength-duration data [19–21].

It is worth emphasizing that the order *α* of the fractional passive membrane does not simply alter the effective time constant, but rather the dynamics at multiple time scales. To illustrate, we consider the response of the fractional passive membrane following a step current input, to characterize the sub-threshold response in a neuron or other excitable cell. The membrane potential *V*_*m*_(*t*) time course, given by Eq 7, is shown as a function of time for different values of *α* in Fig 1C. For small *α*, the membrane *initially charges faster*, compared with the exponential solution when *α* = 1, following the current step input. Subsequently, *V*_*m*_(*t*) approaches the steady-state voltage more slowly. Thus, in this study, we investigate to what extent the sub-threshold dependence on *α* will also influence the super-threshold response and the properties of the active neuron and interacting neurons.

### Fractional-order membrane patch model

With sufficient numerical tools to proceed, we present here the fractional-order Hodgkin-Huxley (fHH) model of the neuron:

$$\begin{array}{c} C_{m}^{\alpha }\frac{d^{\alpha }v}{dt^{\alpha }}=I\left(t\right)-g_{Na}m^{3}h\left(v-E_{Na}\right)-g_{K}n^{4}\left(v-E_{K}\right)-g_{L}\left(v-E_{L}\right) \end{array}$$ (17a)

$$\begin{array}{c} m^{\prime }\left(t\right)=\alpha_{m}\left(1-m\right)-\beta_{m}m \end{array}$$ (17b)

$$\begin{array}{c} h^{\prime }\left(t\right)=\alpha_{h}\left(1-h\right)-\beta_{h}h \end{array}$$ (17c)

$$\begin{array}{c} n^{\prime }\left(t\right)=\alpha_{n}\left(1-n\right)-\beta_{n}n, \end{array}$$ (17d)

where *v* = *V*_*m*_−*V*_*rest*_ represents the membrane potential *V*_*m*_ relative to the resting potential *V*_*rest*_, *m*, *h*, and *n* are the sodium current *I*_*Na*_ activation, *I*_*Na*_ inactivation, and potassium current *I*_*K*_ activation gating variables, respectively, *I*(*t*) is a time-dependent applied stimulus, the prime denotes a first-order derivative with respect to time, and *d* ^*α*^ *v*/*dt* ^*α*^ refers to the fractional derivative in the Caputo sense (since we specify integer-order initial conditions).

#### Numerical integration

We can use Eq 15 to integrate the entire system (Eq 17) using a specified value for *α* for integration of Eq 17a and a value of 1 for Eqs 17b–17d. In practice, summation of the previous history becomes computational expensive for long simulations, and thus integration of Eqs 17b–17d was performed via forward Euler coupled with the Grunwald-Letnikov integration scheme in MATLAB (Mathworks, Inc.), using of a time step of Δ*t* = 10^−4^−10^−3^ ms. As *α* decreases, we found that a smaller integration time step Δ*t* is required for numerical stability, which leads to significantly longer simulation times. Maximum time steps and simulation times are shown as functions of *α* in S1 Text (Fig. B in S1 Text). Standard parameters and initial conditions (Table A in S1 Text), and gating equations are given in S1 Text, and simulation code is provided in S1 Code.

Numerical integration using the standard forward Euler method, i.e., for *α* = 1, the membrane potential *v* at time *t*_*n*_, *v*(*t*_*n*_), is a function of state variables at time *t*_*n*−1_, whereas for 0 < *α* < 1, *v*(*t*_*n*_) is a function of all of prior history of the membrane potential due to capacitive memory. Using the numerical scheme in Eq 15, we can directly characterize the influence of capacitive memory by defining a *voltage memory trace*, following the similar approach of Teka et al. [22], that is the weighted sum of previous values of membrane potential, excluding the immediate previous term,

$$\begin{array}{c} v_{mem}\left(t_{n+1}\right)=\sum_{k=1}^{n}c_{k+1}^{\alpha }v\left(t_{n-k}\right),\;\;n\geq 1 \end{array}$$ (18)

where we define *v*_*mem*_(*t*_0_) = *v*_*mem*_(*t*_1_) = 0.

### Spikes triggered by a brief stimulus pulse

#### Spike properties

**Fig 2:**
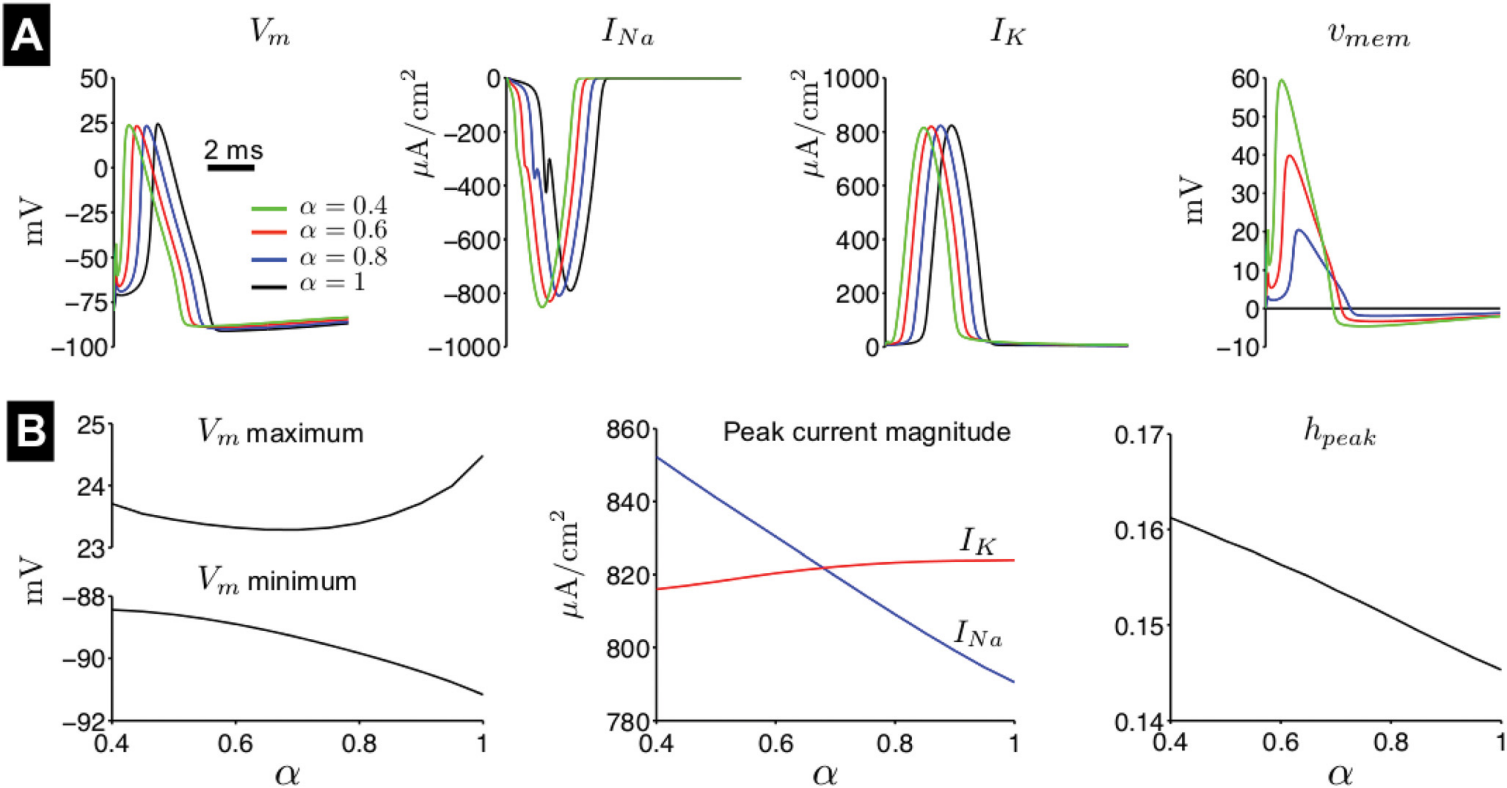
Properties of the fractional-order Hodgkin-Huxley spike. (A) The membrane potential *V*_*m*_, sodium current *I*_*Na*_, potassium current *I*_*K*_, and voltage memory trace *v*_*mem*_ are shown as a function of time, for different values of fractional-order *α*. (B). *V*_*m*_ maximum and minimum (left), *I*_*Na*_ and *I*_*K*_ peak current magnitude, and *h*_*peak*_ (the sodium inactivation gating variable at the time of peak *I*_*Na*_ current) are shown as a function of *α*. Spikes are elicited by a brief 0.1-ms duration, 1.5x threshold stimulus.

We first characterize the influence of the fractional-order *α* on the properties of the neuronal spike in response to a brief stimulus pulse (Fig 2). For small *α*, *V*_*m*_ is depolarized rapidly, in fact faster than sodium current (*I*_*Na*_) activation, such that *V*_*m*_ subsequently repolarizes until sufficient time has passed that *I*_*Na*_ activation triggers a full depolarization and the spike upstroke (Fig 2A, red and green traces). As a consequence of the rapid membrane depolarization, the spike peak is earlier for small *α*. However, once triggered, there are only small differences in the properties of the spike and ionic currents. Sodium current *I*_*Na*_ exhibits the largest dependence on *α*: as *α* decreases, the peak *I*_*Na*_ magnitude increases (Fig 2B). The *I*_*Na*_ time course is also slightly modified. We found that the increase in *I*_*Na*_ peak magnitude was primarily due to a decrease in *I*_*Na*_ inactivation, i.e., the *I*_*Na*_ inactivation gate at the time of the current peak, *h*_*peak*_, increases as *α* decreases.

The magnitude of the voltage memory trace *v*_*mem*_ greatly increases and is non-zero over a longer time period as *α* decreases, demonstrating a larger influence on membrane potential dynamics as expected. Less prominent effects include a small decrease in the potassium current (*I*_*K*_) peak magnitude, the *V*_*m*_ maximum (spike peak), and the *V*_*m*_ hyperpolarization overshoot (an increase in *V*_*m*_ minimum), such that the spike amplitude is reduced (Fig 2B).

#### Post-spike refractoriness

**Fig 3:**
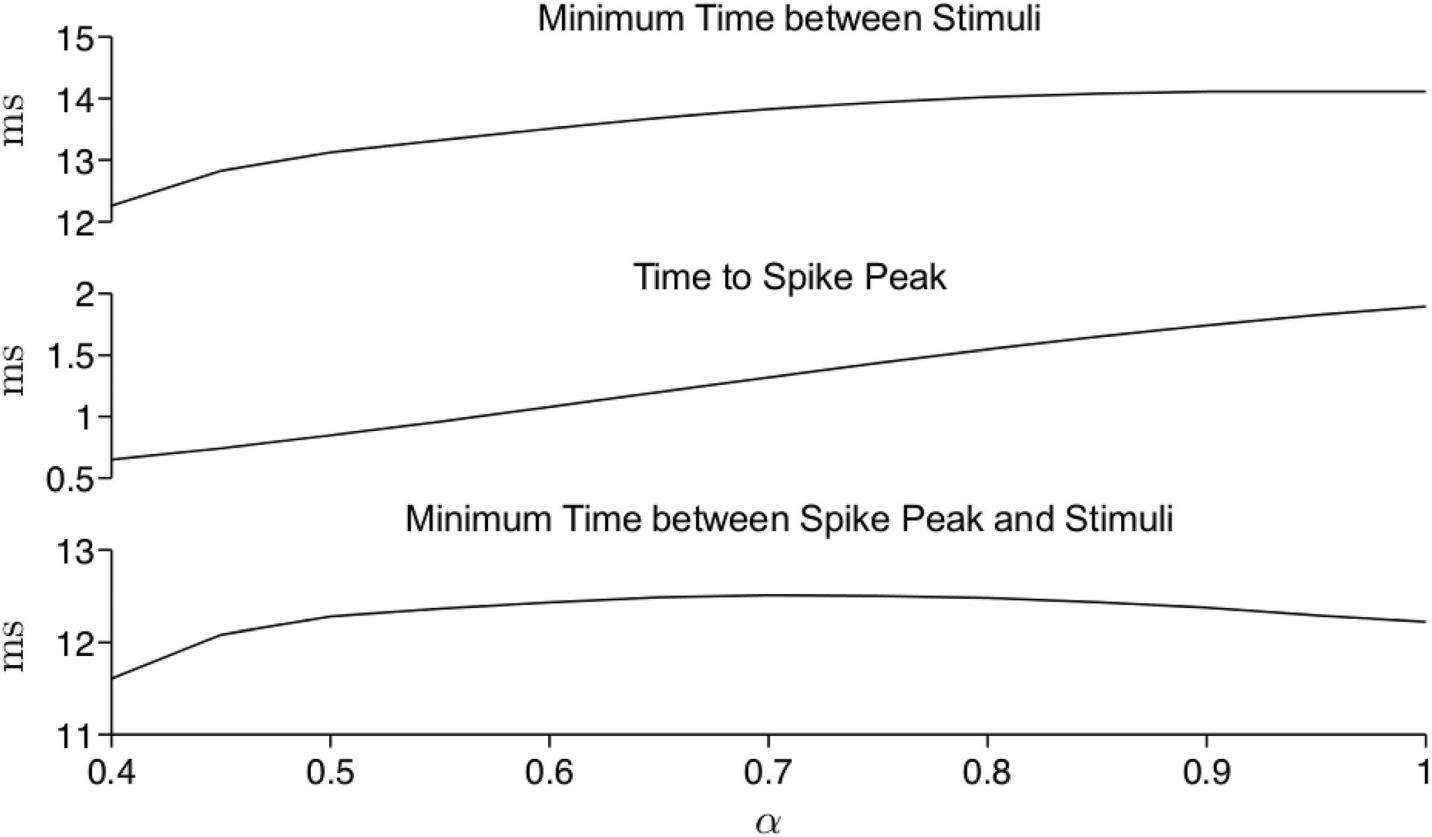
Refractoriness in the fractional-order Hodgkin-Huxley model. The minimum time period between stimuli (top), the time to the spike peak (middle), and their difference (bottom) are shown as a function of fractional-order *α*. Spikes are elicited by a brief 0.1-ms duration, 1.5x threshold stimulus.

To characterize refractoriness following a spike, we measure the minimum time period between stimuli for which two spikes could be triggered by a brief 0.1-ms stimulus (Fig 3). We found that this minimum time decreased as *α* decreased. However, after accounting for the earlier spike peak (as seen in Fig 2A), the difference in refractoriness as *α* varied was small: the time between the spike peak and the subsequent stimuli varied less than 1 ms for *α* between 0.4 and 1. This is likely due to the fact that refractoriness is primarily a consequence of the sodium current *I*_*Na*_ recovery from inactivation, which is minimally influenced by the fractional-order when the stimulus is brief.

### Spikes triggered by a constant stimulus

#### Repetitive firing in the fractional-order neuron model

**Fig 4:**
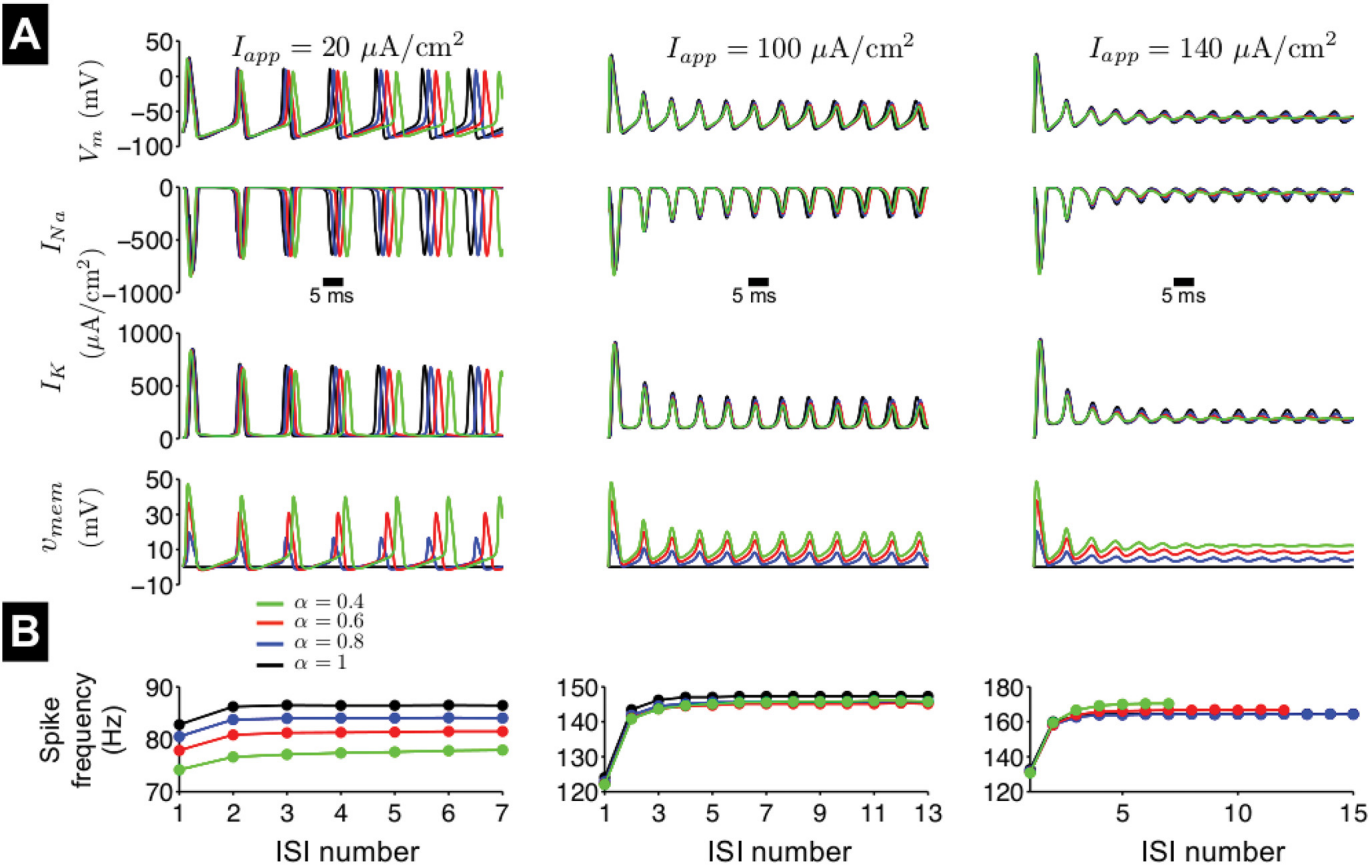
Repetitive firing in the fractional-order Hodgkin-Huxley model. (A) The membrane potential *V*_*m*_, sodium current *I*_*Na*_, potassium current *I*_*K*_, and voltage memory trace *v*_*mem*_ are shown as a function of time in response to a constant applied current, *I*_*app*_ = 20 (left), 100 (middle), and 140 (right) *μ*A/cm^2^, for different values of fractional-order *α*. (B) The instantaneous spike frequency is shown as a function of the interspike interval (ISI) number for different values of *α*.

It is well-established in the classical Hodgkin-Huxley model (i.e., *α* = 1), the neuron will repetitively fire or spike in response to a constant applied stimulus *I*_*app*_ ∈ [*I*_1_, *I*_2_], where *I*_1_ and *I*_2_ represent critical values discussed below [23]. We next investigate to what extent fractional-order *α* alters the properties of repetitive firing for a constant stimulus. We show the membrane potential *V*_*m*_, ionic currents *I*_*Na*_ and *I*_*K*_, and voltage memory trace *v*_*mem*_ as functions of time for a constant applied current, for different values of *I*_*app*_ and *α*, during a 100-ms duration simulation (Fig 4A).

We found a complex time-varying relationship between *α*, *I*_*app*_, and the spike frequency and amplitude. For small values of *I*_*app*_ (Fig 4, left), spike frequency clearly decreases as *α* decreases. Spike amplitude and peak *I*_*K*_ are reduced and peak *v*_*mem*_ is greatly increased, while peak *I*_*Na*_ is slightly increased. The instantaneous spike frequency increases as a function of the interspike interval (ISI) number for all values of *α* and approaches an asymptotic value after 2–3 intervals (Fig 4B). For a given value of *α*, the increase between the first and final interval is small, approximately 5 Hz.

For an intermediate value of *I*_*app*_ (Fig 4, middle), spike frequency decreases to a much smaller extent as *α* decreases. For all values of *α*, spike amplitude is reduced, such that the increase in *v*_*mem*_ as *α* decreases is mitigated. Both peak ionic current magnitudes decrease as *α* decreases. As before, the instantaneous spike frequency increases as a function of ISI number, approaching an asymptotic value after 4–5 intervals. The increase between the first and final interval is larger, approximately 20 Hz. For a larger value of *I*_*app*_ (Fig 4, right), for *α* = 1, small amplitude oscillations or spikes persist, following stimulus onset. However, as *α* decreases, spike amplitude decreases to 0, such that the neuron no longer fires, and *V*_*m*_ is held at an elevated level, a phenomenon known as excitation block.

The mechanisms underlying the dependence of spiking properties and excitation block on *α* are complex and can be generally explained as follows: Following stimulus onset, although peak *I*_*Na*_ increases during the first spike, rapid membrane polarization reduces the time available for *I*_*Na*_ recovery from *inactivation* following the first triggered spike, which reduces peak *I*_*Na*_ current and peak membrane potential *V*_*m*_ during the subsequent spike. This, in turn, reduces *I*_*K*_ *activation* and peak *I*_*K*_ current. Reduced *I*_*K*_ current causes a less hyperpolarized *V*_*m*_ overshoot, which reduces the magnitude of leak current *I*_*L*_, which is depolarizing at this phase of the spike. Reduced *I*_*L*_ decreases the rate of interspike *V*_*m*_ depolarization and thus subsequently reduces spike frequency. Capacitive memory further reduces the *V*_*m*_ depolarization rate, as the voltage memory trace *v*_*mem*_ more heavily weighs the hyperpolarized *V*_*m*_ values during the preceding overshoot. For larger values of *I*_*app*_, over several spikes, the reduced peak *V*_*m*_ also reduces *I*_*Na*_ *activation*, which in turn further reduces *I*_*Na*_ peak current. These feedback interactions are self-limited, and spike and ionic current characteristics stabilize after several spikes, resulting in spike frequency and amplitude approaching asymptotic values. By averaging over time periods that include the neuron at rest and the hyperpolarization overshoot, the voltage memory trace *v*_*mem*_ effectively “dampens” changes in the membrane potential. Even though the magnitude of *v*_*mem*_ decreases as *I*_*app*_ increases, the magnitude of the ionic currents are decreased to a larger extent, such that capacitive memory is more influential for large values of *I*_*app*_. For a larger value of *I*_*app*_, small fractional-order *α* may reduce the spike amplitude sufficiently and result in excitation block.

**Fig 5:**
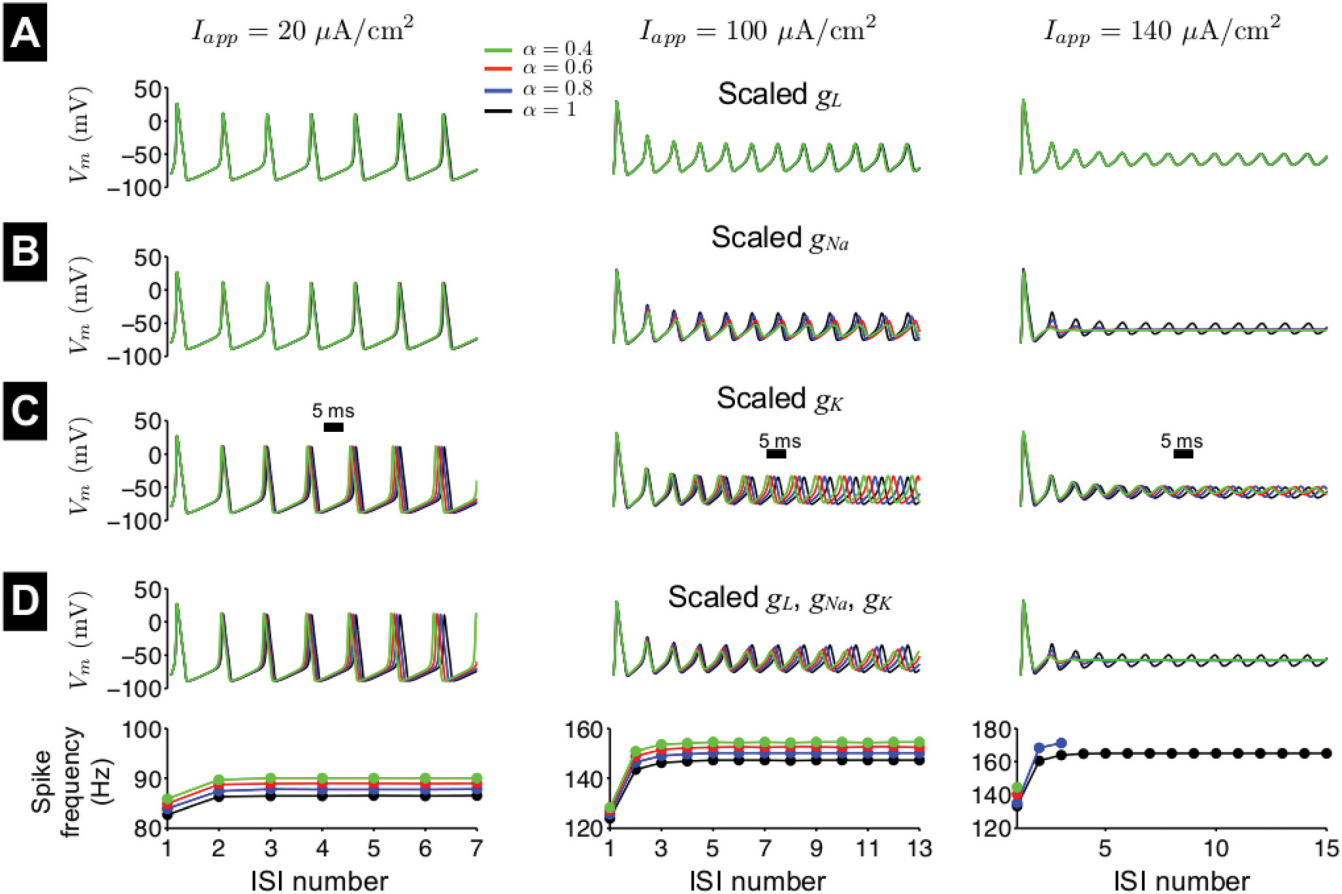
Repetitive firing in the first-order Hodgkin-Huxley model with scaled ionic current conductances. The membrane potential *V*_*m*_ is shown as a function of time in response to a constant applied current, *I*_*app*_ = 20 (left), 100 (middle), and 140 (right) *μ*A/cm^2^, for different values of fractional-order *α*. Sodium, potassium, and leak conductances, *g*_*Na*_, *g*_*K*_, and *g*_*L*_, respectively, are scaled, individually (A-C) and combined (D, top), such that peak current measurements are equivalent to values for particular value of *α*, as described in the text. (D, bottom) The instantaneous spike frequency is shown as a function of the interspike interval (ISI) number for different values of *α*.

**Fig 6:**
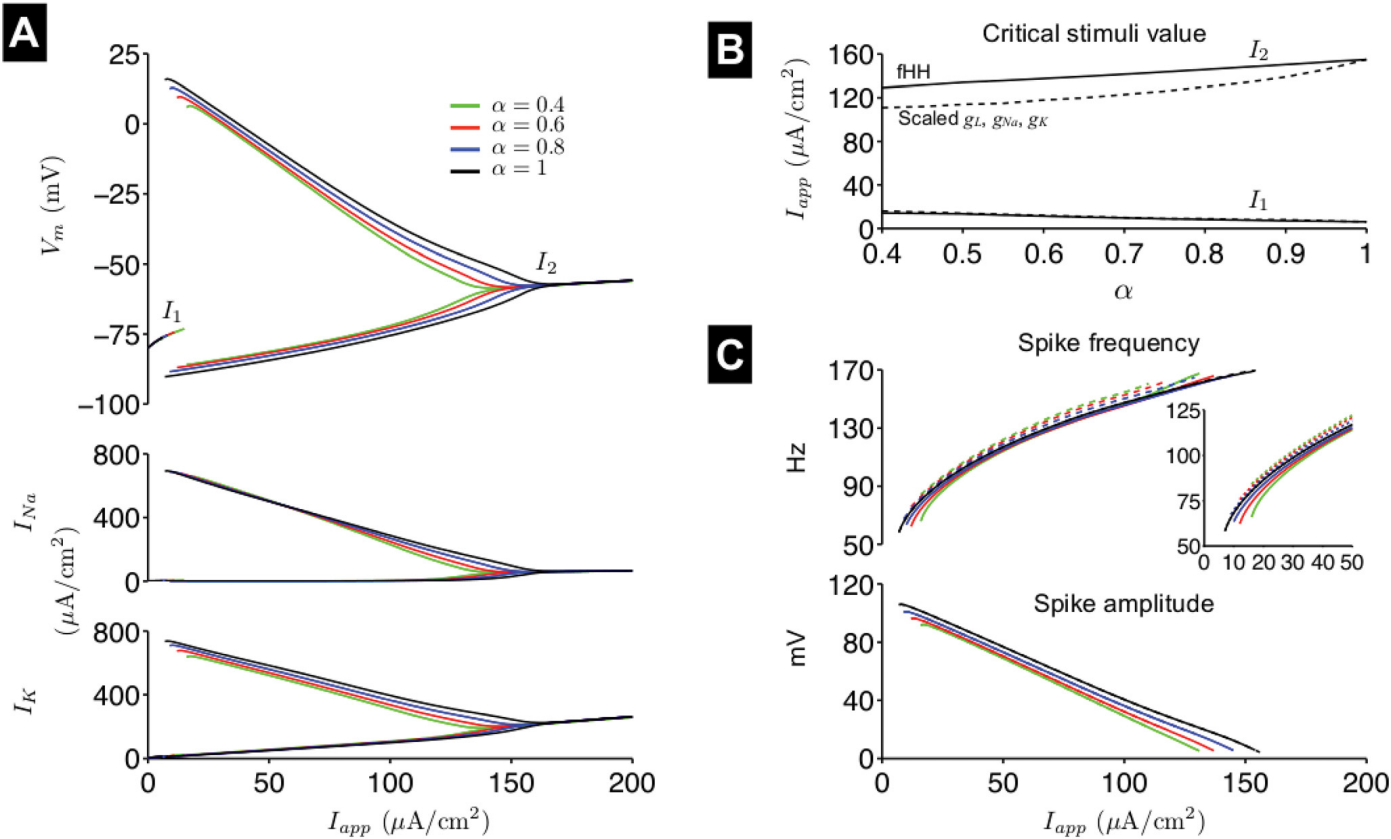
Spike properties in the fractional-order Hodgkin-Huxley model. (A) Bifurcation diagram of *V*_*m*_, sodium current *I*_*Na*_, and potassium current *I*_*K*_, showing steady-state values and limit cycle maximum and minimum, as a function of the applied current *I*_*app*_, for different values of *α*. (B) The critical values denoting *I*_*app*_ lower and upper limits for spiking (Hopf bifurcations), *I*_1_ and *I*_2_, respectively, are indicated (fractional-order Hodgkin-Huxley model (fHH), solid lines). (C) The spike frequency (top) and amplitude (bottom) are shown as a function of *I*_*app*_ and *α*. In B and C, values for *I*_1_, *I*_2_, and spike frequency and amplitude are shown for the first-order model with scaled conductances for comparison (dashed lines, see Fig 5 and main text for more details). In the bottom panel of C, the solid and dashed lines are nearly identical.

To further determine if the dependence of spiking properties on *α* is primarily a consequence of the fractional passive membrane dynamics or the subsequent modulation the ionic currents, both passive and active, we run simulations in which we assume first-order *V*_*m*_ dynamics but also scale the ionic current *I*_*L*_, *I*_*Na*_, and *I*_*K*_ conductances, *g*_*L*_, *g*_*Na*_, and *g*_*K*_, respectively, such that the peak current magnitudes are equivalent to the values for a given *α* in the fractional-order model, i.e., the asymptotic current peak *I*_*Na*_ and *I*_*K*_ values in Fig 4A. Peak *I*_*L*_ magnitude is measured as the maximum depolarizing current, since *I*_*L*_ is both depolarizing and hyperpolarizing. For example, as *α* is decreased in this scaled ionic conductance first-order model, *g*_*K*_ is decreased to account for the smaller peak *I*_*K*_ magnitude. For *I*_*app*_ = 20 *μ*A/cm^2^, *g*_*Na*_ is slightly increased, while for *I*_*app*_ = 100 *μ*A/cm^2^, *g*_*Na*_ is decreased. We investigate the influence of scaling the ionic current conductances both individually and combined (Fig 5). Scaling the leak conductance *g*_*L*_ had minimal influence on spike frequency or amplitude for all values of *I*_*app*_, due to its small amplitude relative to the other currents (Fig 5A). For small *I*_*app*_, scaling the sodium conductance *g*_*Na*_ had minimal influence (Fig 5B). For intermediate *I*_*app*_, scaling *g*_*Na*_ does reduce spike frequency and amplitude, however to a larger extent than observed in the fractional-order neuronal model. For a larger *I*_*app*_, scaling *g*_*Na*_ also leads to excitation block, however with a different dependence on *α* as in the fractional-order model (also see Fig 6B and 6C). For a given value of *I*_*app*_, scaling *g*_*K*_ does not alter spike amplitude and further, increases spike frequency, the opposite effect as observed in the fractional-order model (Fig 5C). When all of the conductances are scaled, the collective influence is that spike frequency increases, not decreases, as the value of *α* decreases (Fig 5D), demonstrating that simply scaling the magnitude of ionic currents does not reproduce the influence of fractional-order *V*_*m*_ dynamics. Interestingly, scaling all three conductances does fairly accurately reproduce the reduction in spike amplitude as *α* decreases.

In Fig 4, we demonstrate that spike amplitude and frequency approach asymptotic values after several spikes during a constant stimulus. Therefore, we next investigated how these asymptotic values varied for different values of *α* and *I*_*app*_. In the classical Hodgkin-Huxley model, as the applied current magnitude *I*_*app*_ increases, the resting potential gradually increases until *I*_*app*_ = *I*_1_ (a subcritical Hopf bifurcation), a critical value at which the neuron fires repetitively. As *I*_*app*_ increases further, spike frequency increases while the spike amplitude decreases, until *I*_*app*_ = *I*_2_ (a supercritical Hopf bifurcation), a critical value for excitation block.

The value of *α* will not influence the steady-state of the system; however *α* can alter the system stability [24] and thus may influence the values for *I*_1_ and *I*_2_ and the range of *I*_*app*_ values for which the neuron spikes. In Fig 6A, we show the steady state for a resting (stable) system and maximum and minimum values for a spiking (unstable) system, for *V*_*m*_, and ionic currents *I*_*Na*_ and *I*_*K*_, as a function of *I*_*app*_. As *α* decreases, the *V*_*m*_ range is reduced (consistent with the reduced spike amplitude in Fig 4), in conjunction with a reduced range for *I*_*Na*_ and *I*_*K*_. The reduction in *I*_*Na*_ is primarily due to a reduction in the sodium activation gate *m*, while the reduction in *I*_*K*_ is primarily due to a reduction in the *V*_*m*_ driving force, with a smaller influence via reduced potassium activation gate *n*. There is minimal influence of the values on sodium inactivation gate *h* (Fig. C in S1 Text).

In Fig 6B (solid lines), we show *I*_1_ and *I*_2_ as a function of *α*. *I*_1_ and *I*_2_ increase and decrease, respectively, as *α* decreases, such that the *I*_*app*_ range for which the neuron spikes is reduced. In Fig 6C (solid lines), the asymptotic values of the spiking frequency (top) and spike amplitude (bottom), calculated over the final 30-ms of a 100-ms simulation, are shown as a function of *α* and *I*_*app*_. In the original Hodgkin-Huxley model (*α* = 1), as *I*_*app*_ increases, spike frequency increases, while spike amplitude decreases (Fig 6C, black lines). We found this relationship—increasing spike frequency and decreasing spike amplitude, for increasing *I*_*app*_—for all values of *α* (Fig 6C, solid color lines). As observed in Fig 4, for all values of *I*_*app*_, spike amplitude decrease as *α* decreases. For small values of *I*_*app*_, spike frequency decreases as *α* decreases. The reduction in spike frequency is mitigated for larger values of *I*_*app*_ and, in fact, spike frequency increases as *α* decreases for *I*_*app*_ near the critical bifurcation value *I*_2_. We show for comparison (dashed lines in Fig 6B and 6C) measurements of *I*_1_, *I*_2_, and spike frequency and amplitude for the first-order model with all ionic current conductances scaled (Fig 5D). Critical value *I*_1_ is similar in the two models, while *I*_2_ is smaller compared with measurements from the fractional-order model, demonstrating that only accounting for the influence of peak ionic currents underestimates the range of stimuli that result in neuronal spiking. As observed in Fig 5D, the reduction in spike amplitude for decreasing *α* is in close agreement between the two models, as the dashed and solid lines are nearly identical. However, spike frequency in the scaled conductance first-order model increases as *α* decreases, in contrast with the fractional-order model.

## Fractional-order nerve axon model

Expanding in scale beyond the spatially-clamped membrane patch, we next consider a spatially-extended system, a one-dimensional cable, to investigate whether the fractional-order *α* influences the properties of electrical propagation in a nerve axon.

### Sub-threshold Propagation

**Fig 7:**
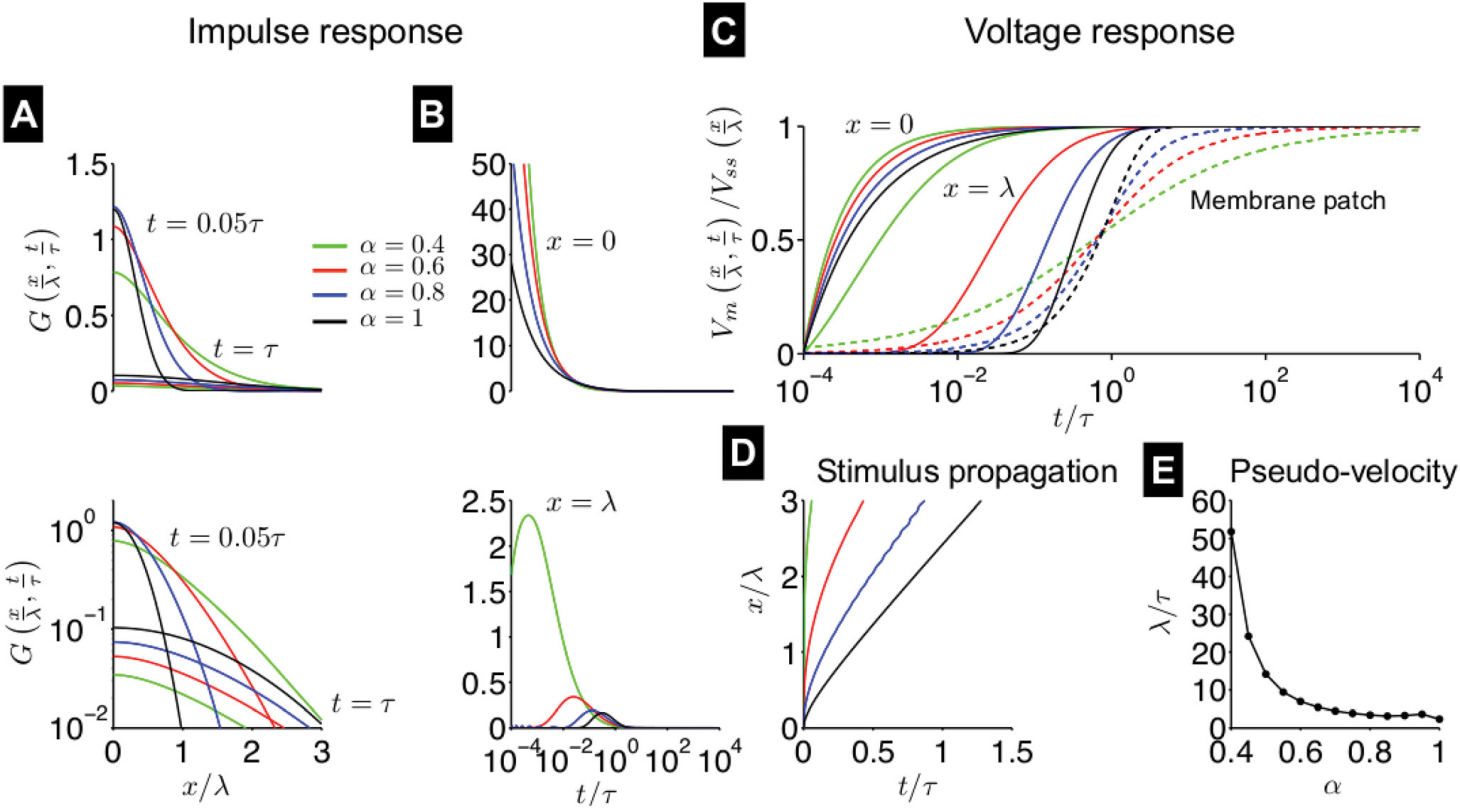
Sub-threshold impulse and voltage response in the passive fractional-order cable equation. (A) The impulse response function *G*(*x*/*λ*, *t*/*τ*) is shown as a function of space *x*, normalized by space constant *λ*, at times *t* = 0.05*τ* and *t* = *τ*, where *τ* is the time constant, on a linear (top) and logarithmic (bottom) scale, for different values of fractional-order *α*. (B) *G*(*x*/*λ*, *t*/*τ*) is shown as a function of normalized time *t*/*τ* at location *x* = 0 (top) and *x* = *λ* (bottom). (C) The normalized voltage response to a current step input at the origin *x* = 0 is shown as a function of normalized time *t*/*τ* at locations *x* = 0 and *x* = *λ*. The voltage response in the membrane patch is shown for comparison (dashed lines, Fig 1C). (D) The normalized position of stimulus propagation *x*/*λ* is shown as a function of normalized time *t*/*τ* (the time at which the normalized voltage response is 0.5) (E) The pseudo-velocity, given by the slope of the stimulus propagation, in units of *λ*/*τ*, is shown as a function of *α*.

As in the previous section, we first investigate the properties of the passive membrane. The fractional-order passive cable equation is given by Eq 6 with the additional of a voltage diffusion term:

$$\begin{array}{c} C_{m}^{\alpha }\frac{\partial^{\alpha }V_{m}}{\partial t^{\alpha }}+\frac{1}{R_{m}}V_{m}=g\frac{\partial^{2}V_{m}}{\partial x^{2}}+I\left(x,t\right), \end{array}$$ (19)

where *g* is a longitudinal cable conductance. Eq 19 can be written in standard form,

$$\begin{array}{c} \tau^{\alpha }\frac{\partial^{\alpha }V_{m}}{\partial t^{\alpha }}=\lambda^{2}\frac{\partial^{2}V_{m}}{\partial x^{2}}-V_{m}+R_{m}I\left(x,t\right), \end{array}$$ (20)

where time constant $\tau^{\alpha }=R_{m}C_{m}^{\alpha }$ and space constant $\lambda =\sqrt{R_{m}g}$. The membrane potential *V*_*m*_(*x*, *t*) can be determined by

$$\begin{array}{c} V_{m}\left(x,t\right)=R_{m}\int_{0}^{t}\int_{-\infty }^{\infty }G\left(x-x^{\prime },t-t^{\prime }\right)I\left(x^{\prime },t^{\prime }\right)dx^{\prime }dt^{\prime }, \end{array}$$ (21)

the convolution of the applied current *I*(*x*, *t*) and *G*(*x*, *t*), the impulse response, scaled by *R*_*m*_. We solve for *G*(*x*, *t*) using an analytical-numerical approach using the Laplace-Fourier transform (see S1 Text), shown in Fig 7A and 7B. At early time points, for small *α*, *G*(*x*, *t*) is more “spread out” in space, while *G*(*x*, *t*) is less spread out at later time points (Fig 7A). At the site of the impulse (*x* = 0) and one length constant away (*x* = *λ*), the impulse is also more spread out in time for small *α* (Fig 7B).

As with the membrane patch, we plot the voltage response to a current step input at the origin (*x* = 0), that is, *I*(*x*, *t*) = *I*_*m*_ *u*(*t*)*δ*(*x*), where *δ*(*x*) is the Dirac delta function. While it is not possible to characterize a “velocity” of the sub-threshold response, since *V*_*m*_ decays to 0 as *x* approaches infinity, we can measure the propagation of *V*_*m*_(*x*, *t*), normalized by the steady-state voltage at that particular spatial location *V*_*ss*_(*x*) (Fig 7C). Membrane polarization is more rapid for small *α* at *x* = 0, as in the membrane patch (Fig 1C, shown here for comparison as dashed lines). Downstream of the current input site, at *x* = *λ*, *V*_*m*_ increases at a much earlier time for small *α*, compared with *α* = 1, that is, the sub-threshold stimulus propagates down the cable faster for small *α*. The position of the stimulus propagation is shown as a function of the time when the normalized voltage response reaches 0.5 (Fig 7D). For small *α*, the stimulus propagation is much faster, and we find that the “pseudo-velocity,” given by the slope of the stimulus propagation position as a function of time, increases as *α* decreases (Fig 7E). For *α* = 1, the pseudo-velocity is approximately 2*λ*/*τ*, consistent a prior analytical calculation for the classical cable equation [25].

### Spike propagation

We next consider the *active* nerve axon and investigate how fractional-order *α* influences spike propagation. As in the previous section, we investigate the response of the neuronal model to a brief stimulus pulse and a constant stimulus. Importantly, simulating the response to both a brief and constant stimulus enables the analysis of fractional-order *α* when capacitive memory may influence spike propagation during a single spike following a resting state and multiple spikes, respectively. The fractional-order Hodgkin-Huxley model can be extended to a cable model by Eq 17a with the addition of a voltage diffusion term:

$$\begin{array}{c} C_{m}^{\alpha }\frac{\partial^{\alpha }v}{\partial t^{\alpha }}=g\frac{\partial^{2}v}{\partial x^{2}}+I\left(x,t\right)-g_{Na}m^{3}h\left(v-E_{Na}\right)-g_{K}n^{4}\left(v-E_{K}\right)-g_{L}\left(v-E_{L}\right), \end{array}$$ (22)

where *g* is the axon conductance. The gating variable dynamics are similarly functions of space and time. In practice, we integrate this system by converting the fractional-order partial differential equation in Eq 22 to a system of fractional-order ordinary differential equations using the method of lines, with a spatial discretization of Δ*x* = 0.5 mm and utilizing the numerical scheme described above (Eq 15). Simulation code is provided in S1 Code.

#### Spike propagation trigged by a brief stimulus pulse

**Fig 8:**
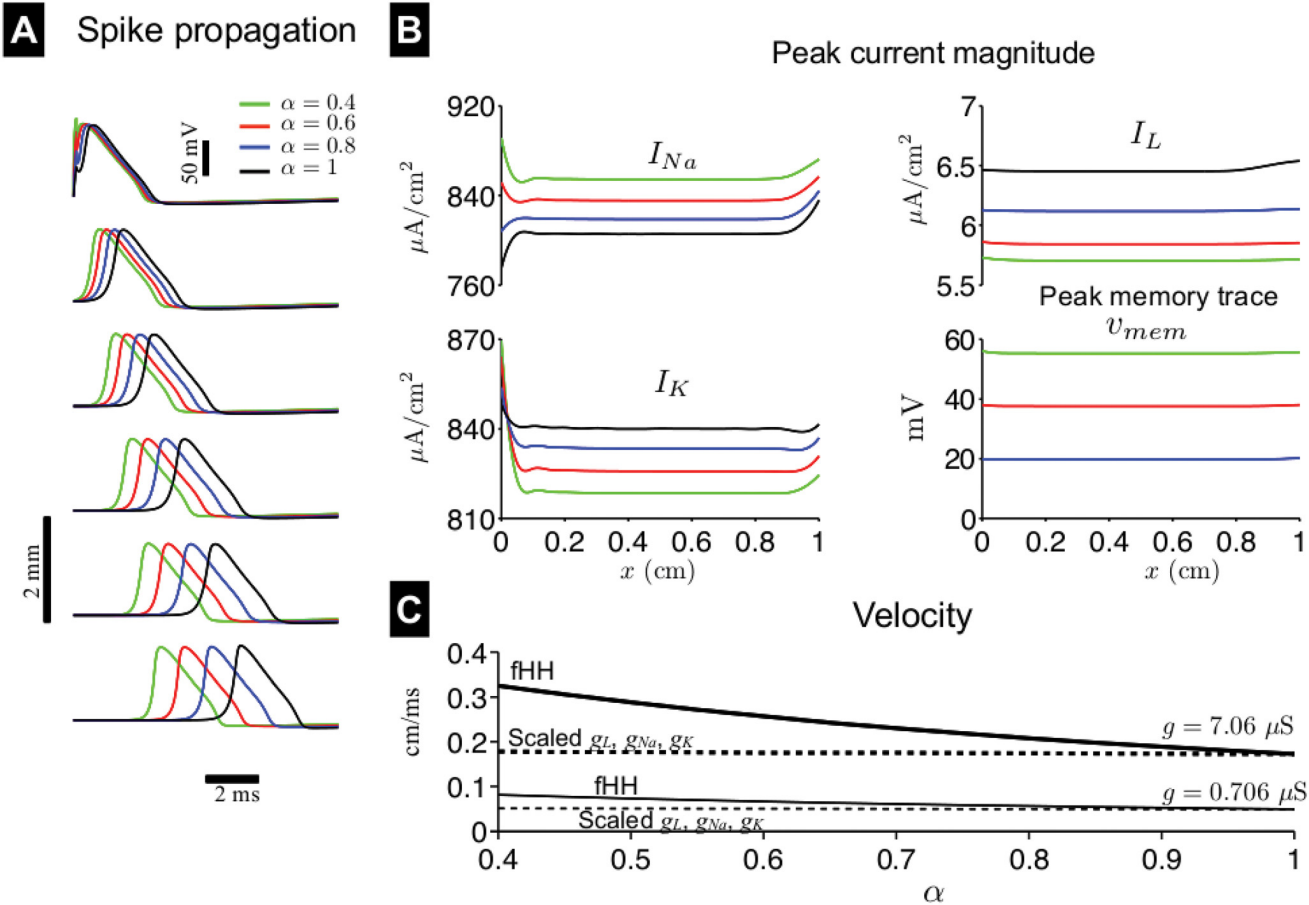
Spike propagation in the fractional-order Hodgkin-Huxley nerve axon following a brief stimulus pulse. (A) A space-time plot of the membrane potential *V*_*m*_(*x*, *t*) is shown for different values of fractional-order *α*. (B) The peak sodium current *I*_*Na*_, potassium current *I*_*K*_, leak current *I*_*L*_, and voltage memory trace *v*_*mem*_ magnitude are shown as a function of position along the cable *x*, for different values of *α*. (C) Spike propagation velocity is shown in the fractional-order Hodgkin-Huxley (fHH) nerve axon, as a function of *α*, for different values of longitudinal conductance *g* (solid lines). Velocity measurements are also shown (dashed lines) for simulations in which the sodium *g*_*Na*_ and potassium *g*_*K*_ conductances are scaled such that peak current measurements are equivalent to values for particular value of *α*. See text for more details. In A and B, axon conductance *g* = 7.06 *μ*S. Propagating spikes are elicited by a brief 0.1-ms duration, 500-*μ*A/cm^2^ stimulus at *x* = 0.

We first consider the influence of fractional-order *α* on spike propagation following a brief stimulus pulse. Representative space-time plots of spike propagation for different values of *α* are shown in Fig 8A. We find that the spike propagates the length of the cable (1 cm) faster as *α* decreases. To determine the mechanism underlying faster spike propagation in the cable, we first measure the peak current magnitudes and voltage memory trace along the length of the cable for different values of *α* (Fig 8B). As in the membrane patch (Fig 2), peak *I*_*Na*_ and *v*_*mem*_ increase and *I*_*K*_ decreases, respectively, as *α* decreases. Propagation velocity is measured by calculating the difference between the spike peak time at *x* = 0.25 and 0.75 cm. We plot the spike propagation velocity as a function of *α* for different axon conductance levels *g* (Fig 8C, solid lines). For both values of *g*, the velocity increases as *α* decreases, consistent with the passive cable; however, the dependence of the velocity on *α* is mitigated as *g* decreases (solid lines, an increase of ∼65% for *g* = 0.706 *μ*S and ∼88% for *g* = 7.06 *μ*S, from *α* = 1 to 0.4).

To determine if the dependence of spike propagation velocity on *α* is primarily a consequence of the properties of the fractional passive cable or the changes in the passive and active ionic currents, as in the previous section, we run simulations in which we assume first-order *V*_*m*_ dynamics but also scale the ionic current *I*_*L*_, *I*_*Na*_, and *I*_*K*_ conductances, *g*_*L*_, *g*_*Na*_, and *g*_*K*_, respectively, such that the peak current magnitudes are equivalent to the values for a given *α* and location *x* (the values in Fig 8B), i.e., we increase *g*_*Na*_ and decrease *g*_*K*_ and *g*_*L*_. In these simulations, spike propagation velocity marginally increases as *α* decreases (Fig 8C, dashed lines, both increase ∼3% from *α* = 1 to 0.4) but not nearly to the extent as in the fractional-order nerve axon model. This result, in conjunction with analysis of the sub-threshold stimulus propagation, suggests that the increase in velocity observed for small *α* is primarily a consequence of the passive cable properties of the axon (Fig 7), and not the modulation of the ionic currents.

#### Spike propagation trigged by a constant stimulus

**Fig 9:**
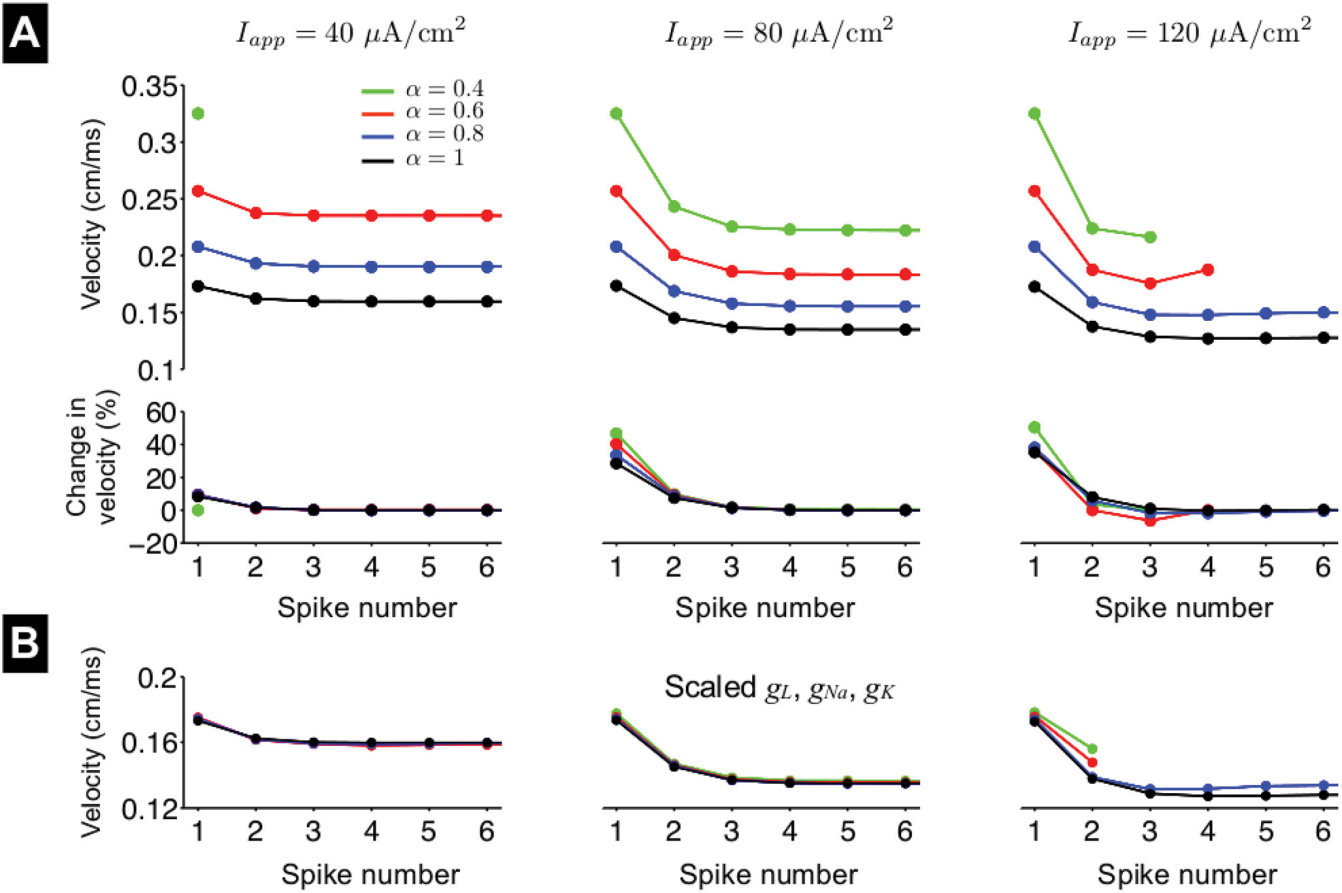
Spike propagation in the fractional-order Hodgkin-Huxley nerve axon during a constant stimulus. (A) Spike propagation velocity (top) and the change in velocity, as a percentage of the final velocity (bottom), are shown as functions of spike number, for different values of fractional-order *α* and applied current amplitude *I*_*app*_. (B) Velocity measurements are shown for simulations in which the sodium, potassium, and leak conductances, *g*_*Na*_, *g*_*K*_, and *g*_*L*_, respectively, are scaled such that peak current measurements are equivalent to values for particular value of *α* and location *x*, as described in the text. Axon conductance *g* = 7.06 *μ*S. Propagating spikes are elicited by a constant stimulus at *x* = 0.

We next investigate the influence of fractional-order *α* on spike propagation during a constant stimulus, in which capacitive memory may alter propagation over successive spikes. In Fig 9A (top), we plot spike propagation velocity as a function of the spike number for different values of *α* and the applied current amplitude *I*_*app*_. In general, spike propagation velocity decreases as a function of the spike number, and in most cases, approaches an asymptotic value after 3–4 spikes. For a small value of *I*_*app*_ (left), velocity decreases to a small extent as spike number increases, and this decrease is approximately the same for all values of *α*. We characterize this decrease by calculating the difference between the velocity for a given spike number and between the final (asymptotic) velocity, as a percentage of the final velocity (Fig 9A, bottom). This small change in velocity is due to the fact that, as in the membrane patch, spike frequency is small for small *I*_*app*_ (Fig 6), such that the time between propagating spikes in the nerve axon is sufficiently long for sodium channel recovery (see Fig. D in S1 Text for further analysis of the relationship between spike frequency and propagation velocity). However, for *α* = 0.4, propagation fails after a single spike, since the sodium channel recovery is insufficient to maintain the fast propagation velocity over many spikes.

For an intermediate *I*_*app*_ (Fig 9, middle, top), velocity decreases to a larger extent as spike number increases, which is a consequence of faster spike frequency and thus less recovery time between spikes. The decrease in the velocity is also larger for smaller *α* (Fig 9, middle, bottom). For larger *I*_*app*_ (Fig 9, right, top), we find that propagation fails after 3 and 4 propagating spikes for *α* = 0.4 and 0.6, respectively, demonstrating that, as in the membrane patch (Fig 6), the range of *I*_*app*_ values for which the axon can sustain repetitive spiking and propagation decreases as *α* decreases.

Interestingly, we note that for all values of *I*_*app*_, the first spike propagation velocity is equal to the velocity following the brief stimulus pulse (Fig 8), demonstrating that capacitive memory does alter spike propagation progressively over several spikes. If we scale the ionic current conductances as before, with the peak *I*_*L*_, *I*_*Na*_, and *I*_*K*_ magnitudes measured over the final 30-ms of a 100-ms simulation (Fig 9B), for most parameters, there is only negligible alteration of the spike propagation velocity. Collectively, these results suggest that the influence of fractional-order *V*_*m*_ dynamics on spike propagation over many spikes is due to fractional-order membrane dynamics and capacitive memory, while the modulation of ionic currents is minimally influential.

## Fractional-order neural network model

Finally, we consider a network of *N* = 50 randomly connected fractional-order Hodgkin-Huxley neurons. We add a synaptic current to the fractional-order Hodgkin-Huxley model (Eq 17a), such that the dynamics of the *i* ^*th*^ neuron are governed by the following equation:

$$\begin{array}{c} C_{m}^{\alpha }\frac{d^{\alpha }v_{i}}{dt^{\alpha }}=I_{i}\left(t\right)-g_{Na}m_{i}^{3}h_{i}\left(v_{i}-E_{Na}\right)-g_{K}n_{i}^{4}\left(v_{i}-E_{K}\right)-g_{L}\left(v_{i}-E_{L}\right)-I_{syn,i}, \end{array}$$ (23)

where the synaptic current is given by the sum of the excitatory and inhibitory synaptic currents,

$$\begin{array}{c} I_{syn,i}=I_{synE,i}+I_{synI,i} \end{array}$$ (24a)

where

$$\begin{array}{c} I_{synE,i}=g_{syn}(\sum_{j\in S_{ex}}s_{ji}\left(v_{j}\right))\left(v_{i}-E_{syn,ex}\right), \end{array}$$ (24b)

$$\begin{array}{c} I_{synI,i}=g_{syn}(\sum_{j\in S_{in}}s_{ji}\left(v_{j}\right))\left(v_{i}-E_{syn,in}\right), \end{array}$$ (24c)

*S*_*ex*_ and *S*_*in*_ are the set of presynaptic neurons with connections to neuron *i*, with excitatory and inhibitory, respectively, synapses, and *s*_*ji*_ is the gating variable for the postsynaptic conductance, an instantaneous, sigmoidal function of the presynaptic cell potential *v*_*j*_ with a threshold *V*_*syn*_ [26], that is

$$\begin{array}{c} s_{ji}\left(v_{j}\right)=\frac{1}{1+\exp[-\left(v_{j}-V_{syn}\right)/k_{syn}]}. \end{array}$$ (24d)

Synaptic current parameters are given in S1 Text (Table B in S1 Text), and simulation code is provided in S1 Code. We have previously studied this type of random neural network [27]. Briefly, synaptic connections were determined as follows: The number of presynaptic connections to the *i* ^*th*^ neuron is drawn from a Gaussian distribution with mean *μ* = 25 and standard deviation *σ* = 2.5, rounded to the nearest whole number. The presynaptic neuron indices *j* are chosen at random from integers [1, *N*]. The type of each synapse, excitatory or inhibitory, is determined at random, such that the probability of an excitatory synapse is on average 0.1. Electrical activity is evoked in the neural network by applying a 40-*μ*A/cm^2^, 50-ms current in 13 randomly selected neurons at time *t* = 0. Simulations of 550-ms duration are performed by integrating the system of fractional-order ordinary differential equations utilizing the numerical scheme described above (Eq 15).

**Fig 10:**
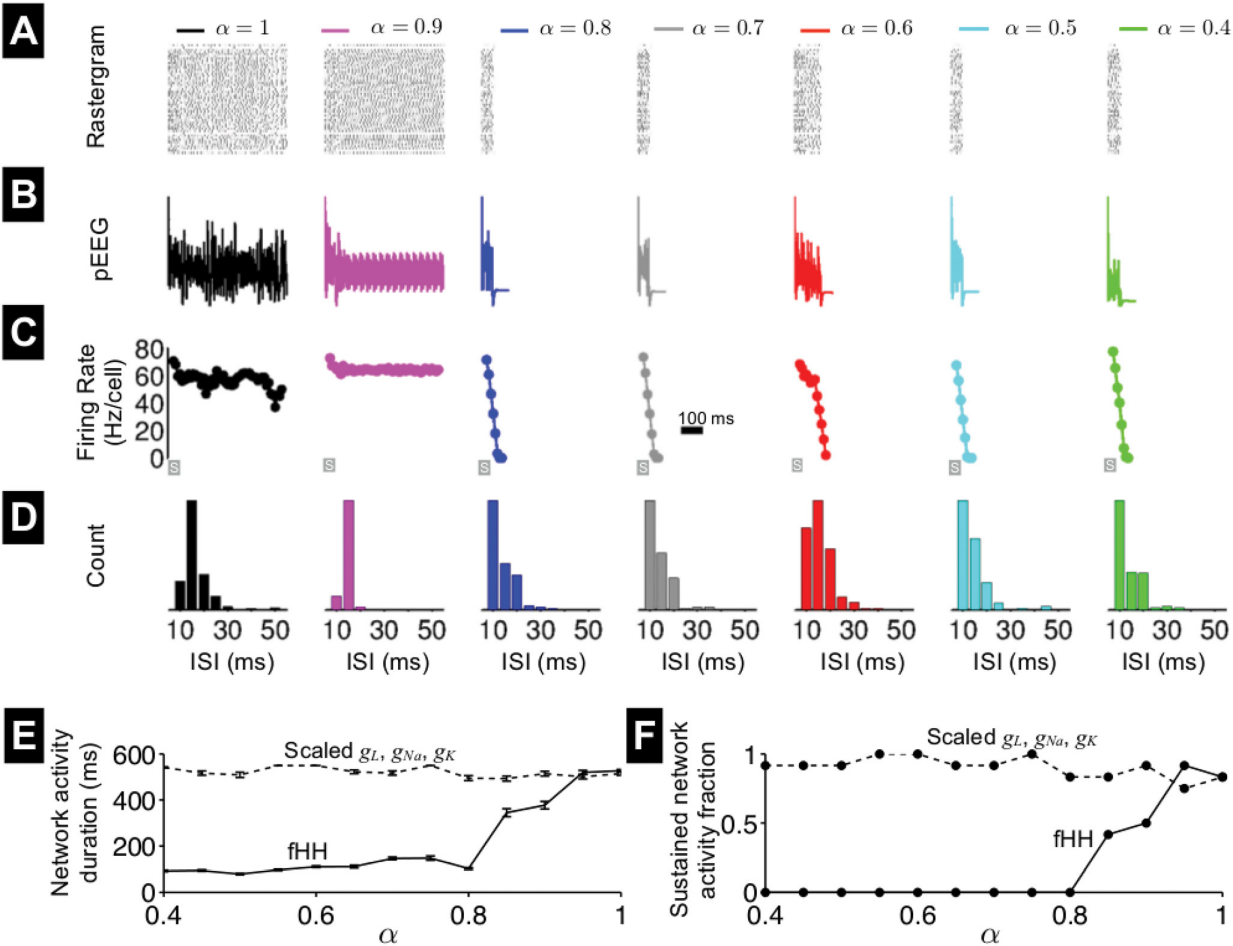
Electrical activity in a fractional-order Hodgkin-Huxley neural network. (A) Rastergram of spikes in the neural network for different values of fractional-order *α*. Synaptic connections and network architecture were identical in all simulations. (B) The pseudo-electroencephalogram (pEEG, Eq 25) and (C) firing rate are shown as functions of time, for the simulations in A. Firing rate is measured in a sliding 50-ms window, with 10-ms steps. (D) Interspike interval (ISI) histograms are shown for each simulation. The gray bar denotes the 50-ms applied stimulus, during which a 40-*μ*A/cm^2^ current was applied to 13 randomly chosen neurons. (E) The mean network activity duration ± standard error of the mean and (F) the fraction of sustained network activity are shown in the fractional-order Hodgkin-Huxley (fHH) neural network, as a function of *α* (solid lines). Network simulations in which the sodium, potassium, and leak conductances, *g*_*Na*_, *g*_*K*_, and *g*_*L*_, respectively, are scaled such that peak current measurements are equivalent to values for particular value of *α*, as described in the text, are shown for comparison (dashed lines). Values in E and F are calculated for 12 network architectures.

We perform simulations of the fractional-order neural network for different values of *α*, in which the network architecture and synaptic connections are identical. The collective activity of the neural network is represented by the rastergram (Fig 10A) and the the pseudo-electroencephalogram (pEEG) [28], given by Λ(*t*),

$$\begin{array}{c} \Lambda \left(t\right)=\frac{1}{N}\sum_{i=1}^{N}v_{i}\left(t\right), \end{array}$$ (25)

the membrane potential averaged over all neurons (Fig 10B). We found that, for *α* = 1 and 0.9, network activity persists for the entire simulation. However, as *α* decreases, network activity self-terminates shortly following the cessation of the 50-ms applied stimulus. The time of network quiescence is not strictly a monotonic function of *α*, as, for example, for this specific network, activity persists longer for *α* = 0.6 compared with 0.8 (see also Fig. E in S1 Text).

Next, we measure the firing rate per cell as a function of time, by counting the total number of spikes in 50-ms sliding windows, with a 10-ms step (Fig 10C). We found that for *α* = 0.9, the firing rate is slightly higher compared with *α* = 1. However, for smaller *α*, the firing rate is initially higher during the stimulus but then sharply decreases and then becomes 0, when the network is quiescent. Histograms of the interspike intervals (ISIs) for the entire network illustrate that, for small *α* and networks that are quiescent following stimulus cessation, there is a higher proportion of short ISI values (Fig 10D). Interestingly, this is contrast with the relationship between spike frequency and *α* in the membrane patch, in which decreasing *α* decreases spike frequency (Fig 6C). Since the synaptic current is essentially a brief stimulus, shorter ISI values for small *α* are a consequence of rapid membrane depolarization, as observed in the membrane patch (Fig 2A), that triggers earlier spike peak times and thus shorter timing between excitatory synaptic current stimuli.

Measured for 12 distinct network architectures, the average duration of network electrical activity (Fig 10E, solid line) and the fraction of networks with sustained electrical activity at the end of the simulation (Fig 10F, solid line) both in general decrease with decreasing *α*. As in previous sections, we run network simulations in which we assume first-order *V*_*m*_ dynamics and scale ionic current conductances (here, scaling to peak current magnitudes for a constant stimulus of *I*_*app*_ = 40-*μ*A/cm^2^ in the membrane patch). In this scaled conductance model, network activity is generally sustained for the entire simulation (Fig 10E and 10F, dashed lines), in agreement with fractional-order *V*_*m*_ dynamics primarily altering network activity by influencing membrane polarization and not modulating ionic currents.

**Fig 11:**
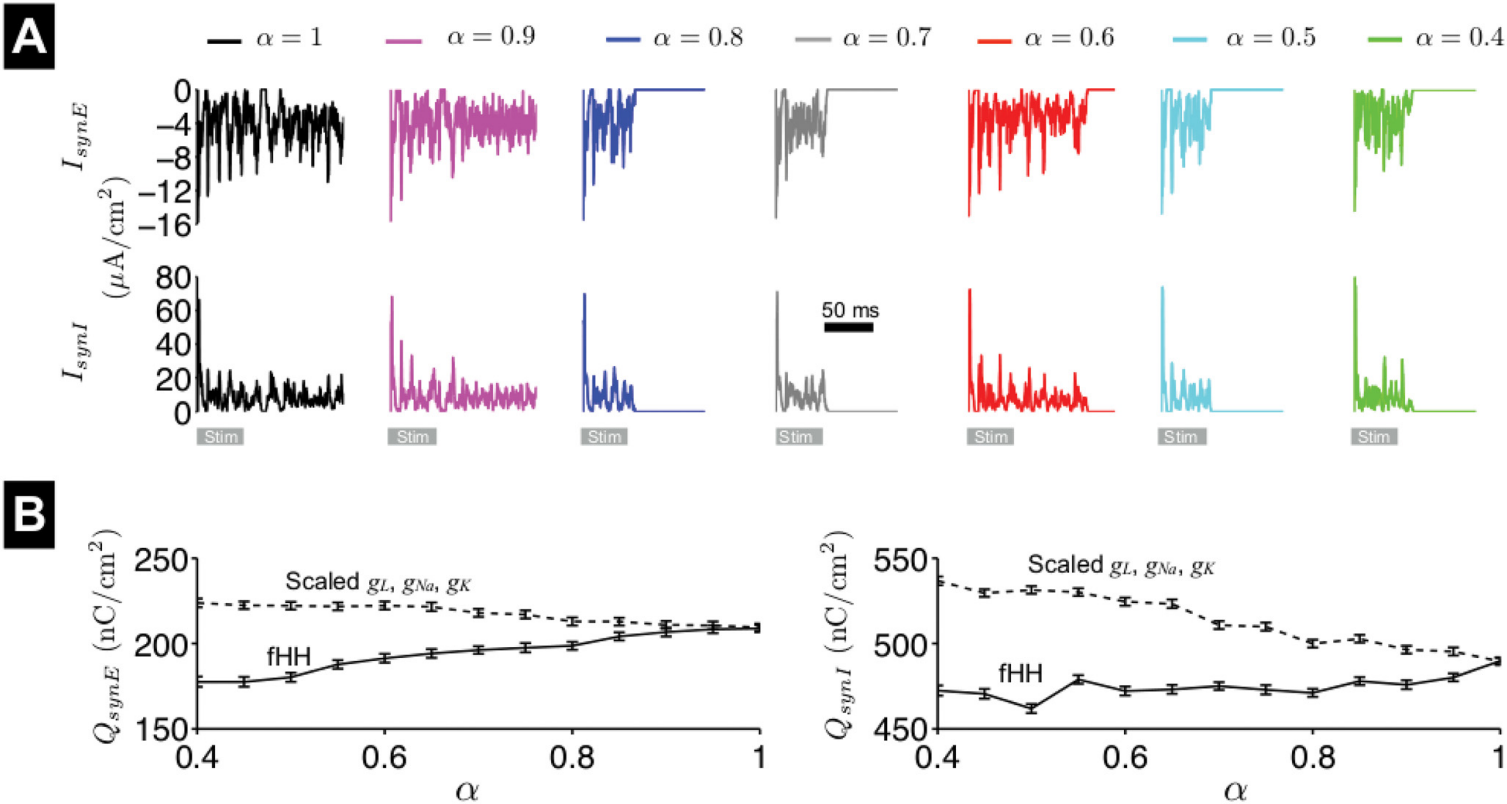
Synaptic activity in a fractional-order Hodgkin-Huxley neural network. (A) The excitatory and inhibitory synaptic currents, *I*_*synE*_ and *I*_*synI*_, respectively, averaged over all network neurons, are shown as a function of time, for different values of fractional-order *α*, for the same network as shown in Fig 10A–10D. The gray bar denotes the 50-ms applied stimulus, during which a 40-*μ*A/cm^2^ current was applied to 13 randomly chosen neurons. (B) The mean of excitatory and inhibitory current charge magnitude, *Q*_*synE*_ and *Q*_*synI*_, respectively, ± standard error of the mean, are shown as a function of *α*, calculated for 12 network architectures (solid lines). Network simulations in which the sodium, potassium, and leak conductances, *g*_*Na*_, *g*_*K*_, and *g*_*L*_, respectively, are scaled such that peak current measurements are equivalent to values for particular value of *α*, as described in the text, are shown for comparison (dashed lines).

Finally, we also investigate the influence of fractional-order *α* on synaptic integration. In Fig 11, we show the excitatory and inhibitory synaptic current, *I*_*synE*_ and *I*_*synI*_, respectively, averaged over all neurons, for the network analyzed in Fig 10A–10D. By close visual inspection, there appears to be a reduction in the synaptic current magnitudes during the applied stimulus (gray bar), as *α* decreases. To quantify this change, we calculated the excitatory and inhibitory synaptic charge, *Q*_*synE*_ and *Q*_*synI*_, the time integral of *I*_*synE*_ and *I*_*synI*_, respectively, during the 50-ms stimulus, and averaged over 12 network architectures (Fig 11B, solid lines). We found that both *Q*_*synE*_ and *Q*_*synI*_ in general decrease as *α* decreases, with the the excitatory synaptic charge *Q*_*synE*_ decreasing to a larger extent.

Despite shorter ISI values and thus more overall spikes, synaptic current and charge are reduced due to the reduced spike amplitude, which reduces both the synaptic gate *s*_*ji*_ (Eq 24d) and the synaptic current driving force. For small *α*, at the cessation of the 50-ms applied stimulus, shorter ISI values result in a larger fraction of network neurons in a refractory state, and combined with reduced excitatory synaptic charge, network activity is more likely to self-terminate. In contrast, in the scaled conductance network model, *Q*_*synE*_ and *Q*_*synI*_ both increase to a small extent as *α* decreases, and these changes negate each other, such that network activity is unaltered (Fig 11B, dashed lines).

## Discussion

### Summary of main findings

In this study, we investigate the properties of fractional-order neuron models and characterize how the fractional-order *α* influences the properties of the neuron, the spatially-distributed axon, and a random network of interconnected neurons. In the passive membrane, we show that, for small *α*, membrane polarization occurs rapidly, and as a consequence, the spike peak is earlier in the neuron model. Further, the bi-directional coupling between *V*_*m*_ and the ionic currents alters the ionic current time course, and the peak sodium and potassium currents increase and decrease, respectively. During a constant applied stimulus, smaller *α* decreases spike amplitude, and for small values of *I*_*app*_, decreases spike frequency. Further, decreasing *α* reduces the range of applied stimulus amplitudes, *I*_*app*_ ∈ [*I*_1_, *I*_2_], that trigger spiking. In the fractional-order passive cable, we show that the impulse response is more spread out in time and space for small *α*, and as a consequence, sub-threshold stimuli propagate faster in the cable. In the nerve axon, spike propagation velocity also increases as *α* decreases, with complex dependence on spike frequency for varying amplitudes of a constant stimulus. Finally, we show in a random network of fractional-order neurons that cessation of neural network activity is more likely as *α* decreases. The fractional-order alters the network activity firing rate; however the dependence on *α* depends on the specific structure of the network.

Importantly, we found that, while the modulation of ionic currents that occur for small values of fractional-order *α* can account for some of the changes in spiking properties, such as spike amplitude, peak ionic current changes cannot account for many important spiking properties, including spike frequency and propagation (and their respective time-dependence during a constant stimulus) and the self-termination of neural network activity. The alteration of these spiking properties primarily arises due to fractional-order passive membrane properties, both in the membrane patch and cable, and the resulting capacitive memory effects.

### Rationale for the fractional-order neuronal model

The original Hodgkin-Huxley model is natural extension of the passive membrane patch model of the parallel resistor-capacitor circuit, with the active sodium and potassium currents represented in the model by time- and voltage-dependent resistors added in parallel. As in the passive membrane patch, ideal capacitive behavior of the cell membrane and thus first-order *V*_*m*_ dynamics are assumed in the original Hodgkin-Huxley model. Motivated by the experimental evidence for non-ideal capacitive behavior in the nerve membrane, as discussed in the Introduction, in this study *V*_*m*_ dynamics are given by a differential equation of fractional order (Eq 17a).

The original Hodgkin-Huxley model assumes that four *I*_*K*_ activation, three *I*_*Na*_ activation, and one *I*_*Na*_ inactivation gates transition independently and that the kinetics of these gates can be represented by reversible isomerization reactions between “not-activated” (“inactivated”) and “activated” (“not-inactivated”) states (Eqs 17b–17d). Although we know that these assumptions are not strictly correct, e.g., *I*_*Na*_ activation and inactivation is not independent and gating transitions often involve multiple “not-activated” states [29], these complications can be easily addressed by incorporating more detailed gating mechanisms, as many studies have done. However, an assumption that is more challenging to address, implicit in this formulation, is that channel gating is a Markov process, i.e., gating transitions only depend on the current system state and do not depend on prior history. While the Markov assumption is generally accepted in the field of ion channel biophysics, some studies have shown that channel gating kinetics present memory in the form of short- and long-term correlations between open- and closed-dwell times [30–33], and that fractal gating kinetics, i.e., transition rates that scale inversely with the duration in a given state, can reproduce such correlations [30, 34]. Mathematical treatments have begun to address the relationship between these dwell time correlations and fractional-order dynamics in theoretical channel models [35, 36] and more generally the relationship between fractional derivatives and fractal dimension [37]. However, to our knowledge, no studies have investigated the relationship between fractional-order gating dynamics and dwell times in a biophysically-detailed gating model. Further work is needed to investigate this relationship. In this study, to specifically address the influence of non-ideal membrane capacitive behavior and fractional-order *V*_*m*_ dynamics, we assume Markovian channel gating, such that gating variable dynamics remain unchanged from the original formulation and assumed first-order.

### Comparison with prior work

To our knowledge, only two prior studies have investigated the fractional-order Hodgkin-Huxley model [38, 39], and both also showed that the fractional-order influenced the time course of the membrane potential *V*_*m*_ and gating variables, including an earlier spike peak time, as we show in Fig 2. Both of these studies also assumed that the gating variables are governed by fractional-order dynamics. As discussed above, ion channel gating memory may be present. However, these studies assume the same fractional-order for *V*_*m*_ and all three gating variables, which we believe is an unjustified modification of the original model given the distinctly different biophysical origin for each source of memory. Further, our analysis is significantly more complete. Here, we characterize the influence of fractional-order on many properties, including spike frequency and amplitude, as well properties of spike propagation in the nerve axon and neural network activity, which are not considered in the aforementioned studies.

A few prior studies have investigated fractional-order *V*_*m*_ dynamics in other excitable cell models. Shi and Wang demonstrated that the fractional-order Morris-Lecar neuron model can exhibit a wider range of bursting behavior than can be reproduced by the original, first-order model [40]. Jun et al. similarly show that fractional *V*_*m*_ dynamics in the Hindmarsh-Rose neuronal model alters spiking patterns and also found a larger applied current threshold for repetitive spiking for smaller fractional-order [41], in agreement with our findings in the Hodgkin-Huxley model (Fig 6B and 6C). Teka et al. demonstrated that fractional dynamics and memory in the leaky integrate-and-fire model altered spike adaptation and firing patterns, including spike latency and interspike variability [22].

Momani et al. studied the coupling of fractional-order neurons modeled using the minimal FitzHugh-Nagumo [42]. However, this study was primarily focused on analysis of numerical techniques. Moaddy et al. studied a neuronal model represented by the fractional passive membrane (analyzed in Fig 1) with the addition of a fractional-order inductor [43]. The fractional-order inductor essentially augments the passive membrane model with an additional slow variable, which is comparable to the FitzHugh-Nagumo model. This study was also primarily focused on numerical techniques; however the authors do find that synchronization of coupled (2 or 3) neurons occurs more quickly as the fractional-order decreased, which appears in agreement with our analysis of larger networks (Fig 10), although the number of networks simulated in our study is too small for a definitive conclusion on synchrony.

While this study and the aforementioned modeling studies focus on fractional-order dynamics for the membrane potential and the influence on neuronal spiking properties, fractional-order dynamics for spiking activity has also been observed experimentally. Lundstrom et al. showed that in response to a sinusoidal stimulus, the firing rate of neocortical pyramidal neurons could be well-approximated by a fractional-order derivative of the input [44]. Anastasio showed that the frequency response of the firing rate, relative to eye position, of premotor neurons in the oculomotor system exhibited fractional-order dynamics, with a phase lag less than 90 degrees (see Fig 1B), suggesting fractional-order dynamics are involved in processing of saccade-related activity [45].

### Physiological implications

The biophysical basis underlying short- and long-term memory is an important area of neuroscience research [46, 47]. The molecular, cellular, and network level mechanisms underlying memory are complex and inevitably involve the interactions between dynamics across a wide range of spatiotemporal scales. An important consequence of fractional-order dynamics is that the membrane potential *V*_*m*_ has memory, in the sense that *V*_*m*_ depends on its entire history from an initial state, albeit with decremental weighting (Fig. A in S1 Text). For fractional-orders in the range considered here, *V*_*m*_ dynamics theoretically may be influenced by history on the order of minutes to hours (see Fig. A in S1 Text). In this study, the small integration time step required for numerical stability (Fig. B in S1 Text) prohibited parameter studies of minute- to hour-long simulations; thus, although our analysis suggests that several important spiking properties, such as spike frequency and propagation velocity, approach asymptotic values on the order of tens of milliseconds, it is possible that dynamics influenced by memory emerge at this longer time scale. Further, in some neural networks, firing rates approach asymptotic values, while in other networks, firing rate is quite variable in time (Fig. E in S1 Text). Thus, the influence of memory on the longer time scale of minutes to hours may be significant in specific network architectures. While the mechanisms underlying neuronal short-term memory are necessarily more complex than memory associated with the cellular membrane dielectric behavior, our study shows that this memory behavior contributes to, or at least modulates, the electrical activity underlying short-term memory. Further work is necessary to thoroughly address this question.

### Practical considerations for neuronal modeling

In this study, we demonstrate that accounting for fractional-order dynamics of the membrane potential alters many properties at the level of the neuron, axon, and network. While we have shown that accounting for fractional-order dynamics may be important, it is not our intention to argue that studies using the “standard” excitable cell model, assuming ideal (first-order) capacitive behavior, are inaccurate. In the neuronal membrane patch following a brief stimulus, the fractional-order *α* primarily influences measures quantitatively, e.g., peak current magnitudes and refractoriness, but did not alter the overall qualitative behavior of the neuron. These properties may be similarly reproduced by altering model parameters.

However, at longer time scales, on the order of tens to hundreds of milliseconds, capacitive memory and fractional-order membrane properties are more significant, such that scaling ionic current conductances does not reproduce important spiking properties, such as spike frequency. Further, at the level of the nerve axon and neural network, we found that fractional-order *V*_*m*_ dynamics can significantly alter emergent properties of the system that are not reproduced by appropriately rescaling ionic current conductances. For example, modulating peak ionic currents does not reproduce the significant increase in propagation velocity in the fractional-order nerve axon model (Fig 8), nor the self-termination of network electrical activity in a random network (Fig 10).

Thus, it is a difficult challenge to determine for which models and settings it is necessary to represent *V*_*m*_ with fractional-order dynamics. Our study suggests that it is important particularly at longer time scales and when accounting for neural membranes that are interconnected via propagation in a nerve axon or synaptic connections in a random network. However, this conclusion may be model-specific (see Limitations section). The use of fractional differential equations to model excitable cells and biological systems in general is presently limited by the complex mathematics involved and limitations on numerical methods. While numerical integration of fractional differential equations is typically more computational expensive compared with integer-order differential equations, advanced integration methods have been recently developed [48–50]. As these methods become more widely used, characterized, and optimized, we expect that modeling of fractional-order excitable cell systems and networks will become more computational feasible, which will allow for further determination of models in which accounting for fractional-order dynamics is important.

### Limitations

The prior studies of fractional-order dynamics, mentioned above, typically focused on minimal neuron models, such as the FitzHugh-Nagumo and Morris-Lecar models, which while valuable, are limited in their biophysical detail. The Hodgkin-Huxley model of the squid giant axon neuron is a classical biophysical model of an excitable cell that is well-characterized, and thus it was a reasonable model to use as a starting point to investigate the influence of fractional-order *V*_*m*_ dynamics on neuronal properties. However, more biophysically-detailed neuronal models relevant to mammalian physiology have been described, incorporating more detailed sodium, potassium, and calcium currents [51, 52]. It may also be important to account for intracellular calcium signaling, specifically stochastic dynamics that can influence electrical activity [53, 54], including spiking frequencies and synaptic transmission.

The properties of the neural networks considered in this study were random; while this type of network provides general insight, the dynamics of activity in networks with a physiological architecture may differ, particularly in the context of disease states such as epileptic seizures [55]. Further work is needed investigate fractional-order dynamics in these more physiologically-realistic neuronal models and networks. Nonetheless, our study provides valuable insight and serves as a reference for investigating fractional-order dynamics in models of neuronal activity.

## Supporting Information

S1 Text Supporting Analysis, Figures, and Tables. Supporting information includes model equations, initial conditions, and parameters, analytical/numerical solutions of passive membrane patch and cable, memory weighting terms, numerical stability analysis, gating variables during spiking, spike frequency and propagation velocity analysis, and neural network firing rate analysis.
(PDF)

S1 Code MATLAB membrane patch, nerve axon, and neural network simulation code. (ZIP)
