# Supplementary material for: Membrane Capacitive Memory Alters Spiking in Neurons Described by the Fractional-Order Hodgkin-Huxley Model
Source: PLoS One. 2015 May 13;10(5):e0126629. doi: 10.1371/journal.pone.0126629 (PMC4430543; doi:10.1371/journal.pone.0126629)
Supplement: S1 Text — Supporting information includes model equations, initial conditions, and parameters, analytical/numerical solutions of passive membrane patch and cable, memory weighting terms, numerical stability analysis, gating variables during spiking, spike frequency and propagation velocity analysis, and neural network firing rate analysis. (PDF) [file pone.0126629.s001.pdf]

S1 Text

Analytical solution of passive membrane via Laplace transform

The membrane potential V_m dynamics of the fractional-order passive membrane is given by the following equation:

$$\tau^\alpha \frac{d^\alpha V_m}{dt^\alpha} + V_m = R_m I_m u(t), \tag{S1}$$

Applying the Laplace transform, given by

$$\widetilde{f}(s) = \mathfrak{L}\{f(t); s\} = \int_0^\infty \exp(-st) f(t) dt, \tag{S2}$$

to Eq. S1 produces

$$\tau^\alpha \left[s^\alpha \widetilde{V}_m(s) - s^{\alpha-1} V_m(0) \right] + \widetilde{V}_m(s) = \frac{R_m I_m}{s} \tag{S3}$$

With a zero initial condition, after rearranging and using partial fractions, we have

$$\widetilde{V}_m(s) = R_m I_m \left[\frac{1}{s} - \frac{s^{\alpha-1}}{s^\alpha + 1/\tau^\alpha} \right]. \tag{S4}$$

We can then use the Laplace transform table in Magin [1] to return to the time domain to show that

$$V_m(t) = R_m I_m \left[1 - E_{\alpha,1}\left(-(t/\tau)^\alpha\right)\right], \tag{S5}$$

the solution given in Eq. 7.

Memory terms in fractional differential equation integration scheme

In Fig. A, panel A, we show the memory terms c_k^α as functions of k for different values of α. For $\alpha = 1$, all c_k^1 terms for $k > 1$ are equal to 0, such that the numerical integration is equivalent to the forward Euler method. For larger integer values of α, all c_k^α terms for $k > \alpha$ are also equal to zero, which is expected based on the finite-difference scheme. For fractional-order α, c_k^α decreases as k increases, as the current value in the numerical integration depends increasingly less on earlier states of the system. However as α decreases, there is an increasingly larger dependence on the system history. For example, the dependence on the $k = 100$ previous state value is weighted approximately 100-fold larger for $\alpha = 0.4$ compared with $\alpha = 0.8$.

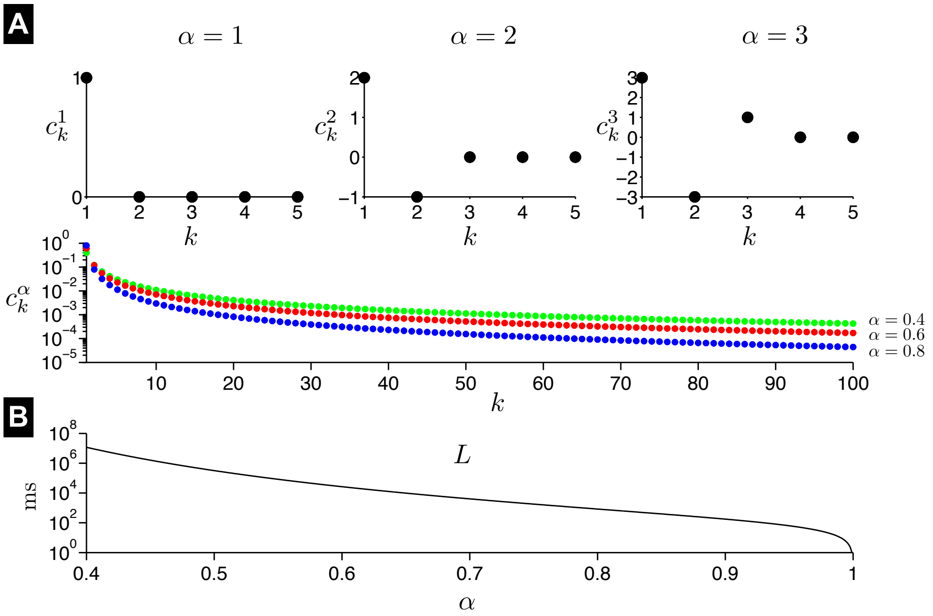

Figure A: **Memory terms in fractional differential equation integration.** (A) The memory terms, c_k^α, used in the fractional differential equation integration scheme (Eq. 15) are shown as a function of k, for integer (top) and fractional (bottom) values of α. (B) The lower bound for necessary "memory" L, for accuracy $\epsilon = 0.01$ mV/ms$^\alpha$ and function bound $M = 100$ mV, is shown as a function of α (Eq. S6).

As the integration time step is made smaller or the simulation duration is made larger, the summation of the entire previous system history becomes increasingly more computationally expensive. One can ask how much of the prior history is necessary for an accurate estimate of the fractional derivative at a given point in time. It has been shown [2] that for a function $y(t)$, if

$y(t) \leq M$, i.e., $y(t)$ is bound by M over some finite time interval, then the necessary "memory" L for accuracy ϵ in the finite difference discretization of ${}_0D_t^{\alpha} y(t)$, is given by

$$L \geq \left(\frac{M}{\epsilon |\Gamma(1-\alpha)|} \right)^{1/\alpha} . \tag{S6}$$

This relationship has a steep dependence on α (Fig. A, panel B). For example, if $M = 100$ mV (a reasonable bound on V_m) and $\epsilon = 0.01$ mV/ms$^{\alpha}$, and $\alpha = 0.5$, 0.7, and 0.9, then $L \geq 3.18 \cdot 10^7$ ms (8.8 hr), $1.1 \cdot 10^5$ ms (1.8 min), and $2.3 \cdot 10^3$ ms (2.3 sec), respectively.

Numerical stability of the Grunwald-Letnikov integration method

We vary the value of α and measure the maximum time step, Δt_{max}, that resulted in a numerically stable integration, using the Grunwald-Letnikov scheme (Eq. 15). Spikes are elicited by a brief 0.1-ms duration, 100-μA/cm^2 stimulus, and simulations are run for a total of 10 ms. Δt_{max} is defined as the largest time step that results in a numerically stable simulation at t =10 ms. In Fig. B, panel A, we show that Δt_{max} decreases as α decreases, such that Δt_{max} is approximately two orders of magnitude smaller for $\alpha = 0.4$ compared with $\alpha = 1$.

Importantly, note that the smaller time step required for smaller α does not simply increase simulation time in a linear fashion, as with the standard Euler method. A smaller time step also requires the summation of more memory terms, and as the simulation duration increases, this summation becomes increasingly more computationally expensive. In Fig. B, panel B, we show that simulation time, for simulation durations of 100 ms, increase steeply as α decreases, when the simulation time step equal to Δt_{max}.

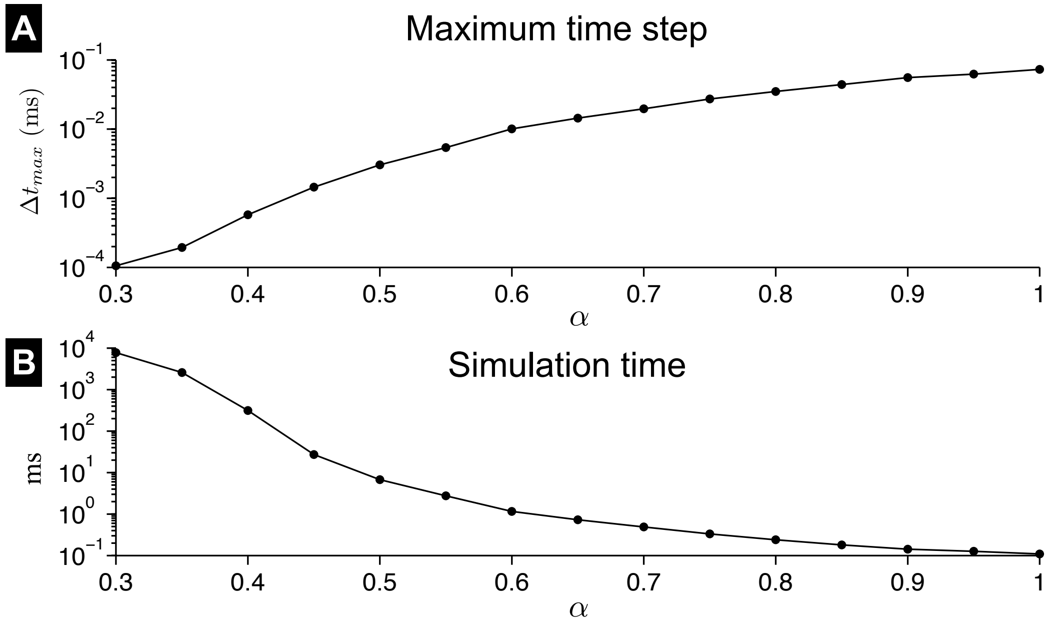

Figure B: **Maximum numerically stable time step.** (A) The largest numerically time step Δt_{max} is shown as a function of fractional-order α. (B) The simulation time, for simulation durations of 100 ms, is shown as a function of α, when the simulation time step equal to Δt_{max}.

Analytic/numerical solution of the fractional cable equation

We follow the same general approach that Mainardi and Pagnini [3] applied to the time-fractional diffusion equation. The impulse response function $G(x,t)$ in Eq. 21 solves the following fractional partial differential equation:

$$\tau^{\alpha}\frac{\partial^{\alpha}G}{\partial t^{\alpha}} = \lambda^{2}\frac{\partial^{2}G}{\partial x^{2}} - G + \delta(x)\delta(t), \tag{S7}$$

where the impulse current input is scaled to normalize the membrane resistance R_m, i.e., $I(x,t) = R_m^{-1}\delta(x)\delta(t)$. Note that $G(x,t)$ is the Green's function or fundamental solution for this fractional-order cable equation. After normalizing Eq. S7 in space and time and rearranging, we have

$$\frac{\partial^{\alpha}G(X,T)}{\partial T^{\alpha}} - \frac{\partial^{2}G(X,T)}{\partial X^{2}} + G(X,T) = \delta(X)\delta(T) \tag{S8}$$

where normalized time $T = t/\tau$ and normalized position $X = x/\lambda$. We next apply the Laplace transform in the temporal dimension (Eq. S2),

$$s^{\alpha}\widetilde{G}(X,s) - s^{\alpha-1}G(X,0) - \frac{d^{2}\widetilde{G}(X,s)}{dX^{2}} + \widetilde{G}(X,s) = \delta(X), \tag{S9}$$

which simplifies to

$$s^{\alpha}\widetilde{G}(X,s) - \frac{d^{2}\widetilde{G}(X,s)}{dX^{2}} + \widetilde{G}(X,s) = \delta(X), \tag{S10}$$

since $G(X,0) = 0$. We next apply the Fourier transform,

$$\widehat{f}(k) = \mathfrak{F}\{f(X);k\} = \int_{-\infty}^{\infty}\exp(-ikX)f(X)dX, \tag{S11}$$

to the spatial dimension, and find

$$s^{\alpha}\widehat{\widetilde{G}}(k,s) + k^{2}\widehat{\widetilde{G}}(k,s) + \widehat{\widetilde{G}}(k,s) = 1 \tag{S12}$$

After rearranging, we have the Fourier-Laplace transform of the Green's function $G(x,t)$:

$$\widehat{\widetilde{G}}(k,s) = \frac{1}{s^{\alpha} + k^{2} + 1} \tag{S13}$$

Using the Laplace transform table in Magin [1], the inverse Laplace transform of Eq. S13 is given by

$$\widehat{G}(k,T) = T^{\alpha-1}E_{\alpha,\alpha}\left(-(k^{2}+1)T^{\alpha}\right), \tag{S14}$$

and then finally find the inverse Fourier transform of Eq. S14,

$$G(X,T) = \frac{1}{2\pi}\int_{\infty}^{\infty}\widehat{G}(k,T)\exp(ikX)dk. \tag{S15}$$

We computed the inverse Fourier transform (Eq. S15) numerically using the MATLAB function `ifft` at a specified instant in time. Note that for $\alpha = 1$, $G(X, T)$ can be analytically solved to arrive at the classic solution of the cable equation, given by

$$G(X, T) = \frac{1}{2\sqrt{\pi T}} \exp(-X^2/(4T)) \exp(-T). \tag{S16}$$

Finally, for application in Eq. 21, we return the numerical solution of Eq. S15 to dimensional form.

Hodgkin-Huxley model equations

The equations governing the dynamics of the gating variables m, h, and n are given by

$$\alpha_m = \frac{2.5 - 0.1v}{\exp(2.5 - 0.1v) - 1} \qquad \beta_m = 4\exp(-v/18)$$

$$\alpha_h = 0.07\exp(-v/20) \qquad \beta_h = \frac{1}{\exp(3 - 0.1v) + 1}$$

$$\alpha_n = \frac{0.1(1 - 0.1v)}{\exp(1 - 0.1v) - 1} \qquad \beta_n = 0.125\exp(-v/80)$$

where voltage $v = V_m - V_{rest}$ is the shifted transmembrane potential, in which the resting potential V_{rest} has been subtracted. The standard parameters for the Hodgkin-Huxley model and synaptic currents are given in Tables A and B.

Table A: **Fractional-order Hodgkin-Huxley model parameters**

Parameter	Definition	Units	Value
g_{Na}	maximum Na^+ current conductance	mS/cm^2	120
g_K	maximum K^+ current conductance	mS/cm^2	36
g_L	maximum leak current conductance	mS/cm^2	0.3
E_{Na}	Na^+ current reversal potential	mV	115
E_K	K^+ current reversal potential	mV	-12
E_L	leak current reversal potential	mV	10.6
V_{rest}	resting potential	mV	-80
C_m^α	fractional-order capacitance	$\mu A \cdot s^\alpha/(V \cdot cm^2)$	1
v_0	initial condition for voltage	mV	0
m_0	initial condition for Na^+ current activation gate	-	0.0529
h_0	initial condition for Na^+ current inactivation gate	-	0.5961
n_0	initial condition for K^+ current activation gate	-	0.3177

Parameters and initial conditions for ionic currents and state variables in the fractional-order Hodgkin-Huxley model.

Table B: **Synaptic current parameters**

Parameter	Definition	Units	Value
g_{syn}	maximum synaptic conductance	mS/cm^2	0.3
$E_{syn,ex}$	excitatory synapse reversal potential	mV	80
$E_{syn,in}$	inhibitory synapse reversal potential	mV	-12
V_{syn}	presynaptic cell potential threshold	mV	50
k_{syn}	threshold parameter	mV	2

Parameters for excitatory and inhibitory synaptic currents in neural network.

Gating variables during spiking

In Fig. C, we show the steady state for a resting (stable) system and maximum and minimum values for a spiking (unstable) system, for V_m, and gating variables m, h, and n, as a function of I_{app}. As discussed in the main text, as α decreases, the V_m range is reduced. This reduction in the V_m range occurs in conjunction with a reduced range for the sodium activation gate m and to a lesser extent the potassium activation gate n. There is minimal influence of the values for the sodium inactivation gate h.

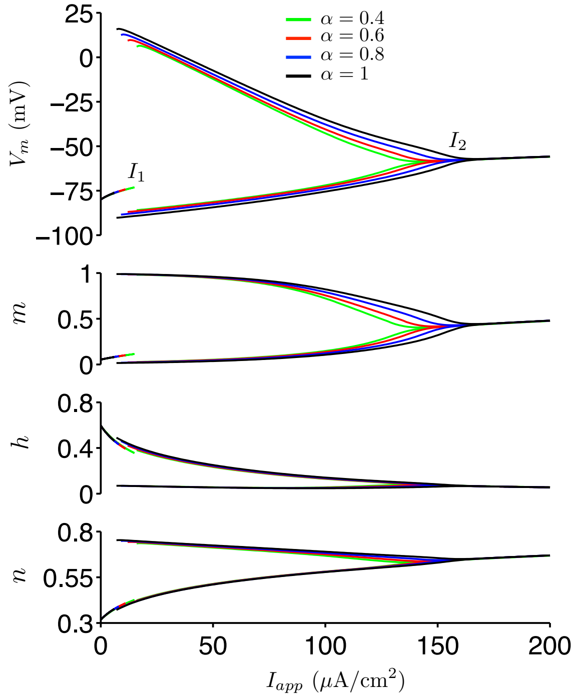

Figure C: **Gating variables during spiking.** Bifurcation diagram of V_m and gating variables m, h, and n, showing steady-state values and limit cycle maximum and minimum, as a function of the applied current I_{app}, for different values of α. The critical values denoting I_{app} lower and upper limits for repetitive firing (Hopf bifurcations), I_1 and I_2, respectively, are indicated.

Relationship between propagation velocity and spike frequency in the nerve axon

In Fig. D, we plot the spike propagation velocity, for spike $i + 1$, as a function of the inverse of the preceding interspike interval i, or spike frequency, at the stimulus site ($x = 0$), for different values of α and I_{app}. Although there is a not clear linear relationship between the spike frequency at the stimulus site and propagation velocity for all parameters, for a given value of α and I_{app}, as the spike frequency increases, the propagation velocity tends to decrease. For small I_{app} and given value of α, spike frequency increases to a small extent over several spikes, and propagation velocity decreases to a correspondingly small extent as well. In contrast, for larger values of I_{app} and a given value of α, spike frequency increases to a larger extent, while propagation velocity decreases to a larger extent.

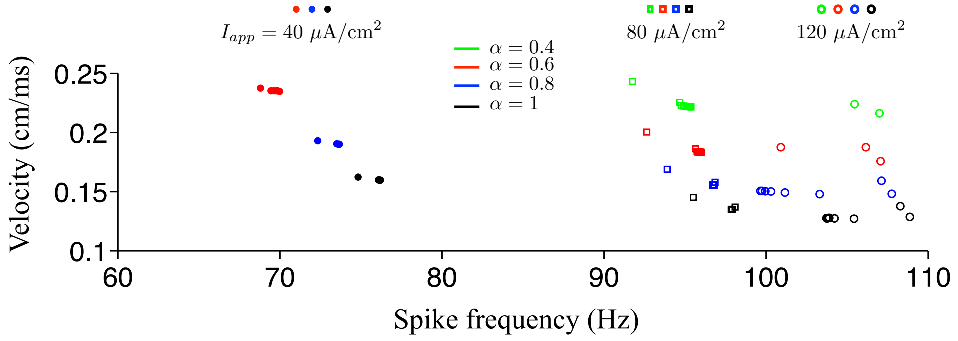

Figure D: **Propagation velocity and spike frequency in the nerve axon.** The propagation velocity for spike $i + 1$ is shown as a function of the instantaneous spike frequency at the stimulus site ($x = 0$), the inverse of the preceding interspike interval i, as a function of the fractional-order α and applied stimulus I_{app}.

Fractional neural network activity

In Fig. E, we show the firing rate as a function of time, for different values of α and 12 network architectures. We find that in many networks, firing rate is increased for $\alpha = 0.9$, compared with $\alpha = 1$, although this is not always the case. In some networks, firing rate remains fairly constant as a function of time, e.g., Network 7, while in other networks, firing rate varies substantially, e.g., Network 10. In all networks, for $\alpha \leq 0.8$, network activity ceases before the end of the simulation, although, as mentioned in the main text, the timing of network quiescence is not a strictly monotonic function of α.

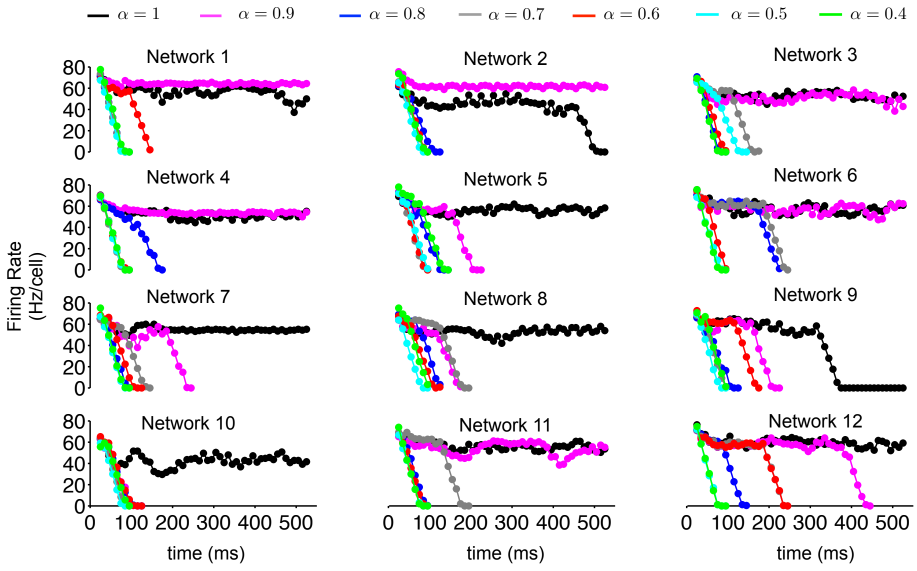

Figure E: **Fractional-order network activity.** The firing rate is shown as functions of time, for different values of fractional-order α and 12 different network architectures. The network analyzed in Figs. 10A-D and 11A is Network 1.

Supporting References

1. Magin RL (2004) Fractional Calculus in Bioengineering, Part 1. Crit Rev Biomed 726 Eng 32: 1-104.

2. Podlubny I (1998) Fractional Differential Equations. An Introduction to Fractional Derivatives, Fractional Differential Equations, to Methods of Their Solution and Some of Their Applications. San Diego, California: Academic Press.

3. Mainardi F, Pagnini G (2003) The Wright functions as solutions of the

time-fractional diffusion equation. Applied Mathematics and Computation 141: 51-62.
